# Kinetic modeling of H_2_O_2_ dynamics in the mitochondria of HeLa cells

**DOI:** 10.1371/journal.pcbi.1008202

**Published:** 2020-09-14

**Authors:** Kassi T. Stein, Sun Jin Moon, Athena N. Nguyen, Hadley D. Sikes

**Affiliations:** 1 Department of Chemical Engineering, Massachusetts Institute of Technology, Cambridge, MA, United States of America; 2 Department of Biological Engineering, Massachusetts Institute of Technology, Cambridge, MA, United States of America; Washington University School of Medicine, UNITED STATES

## Abstract

Hydrogen peroxide (H_2_O_2_) promotes a range of phenotypes depending on its intracellular concentration and dosing kinetics, including cell death. While this qualitative relationship has been well established, the quantitative and mechanistic aspects of H_2_O_2_ signaling are still being elucidated. Mitochondria, a putative source of intracellular H_2_O_2_, have recently been demonstrated to be particularly vulnerable to localized H_2_O_2_ perturbations, eliciting a dramatic cell death response in comparison to similar cytosolic perturbations. We sought to improve our dynamic and mechanistic understanding of the mitochondrial H_2_O_2_ reaction network in HeLa cells by creating a kinetic model of this system and using it to explore basal and perturbed conditions. The model uses the most current quantitative proteomic and kinetic data available to predict reaction rates and steady-state concentrations of H_2_O_2_ and its reaction partners within individual mitochondria. Time scales ranging from milliseconds to one hour were simulated. We predict that basal, steady-state mitochondrial H_2_O_2_ will be in the low nM range (2–4 nM) and will be inversely dependent on the total pool of peroxiredoxin-3 (Prx3). Neglecting efflux of H_2_O_2_ to the cytosol, the mitochondrial reaction network is expected to control perturbations well up to H_2_O_2_ generation rates ~50 μM/s (0.25 nmol/mg-protein/s), above which point the Prx3 system would be expected to collapse. Comparison of these results with redox Western blots of Prx3 and Prx2 oxidation states demonstrated reasonable trend agreement at short times (≤ 15 min) for a range of experimentally perturbed H_2_O_2_ generation rates. At longer times, substantial efflux of H_2_O_2_ from the mitochondria to the cytosol was evidenced by peroxiredoxin-2 (Prx2) oxidation, and Prx3 collapse was not observed. A refined model using Monte Carlo parameter sampling was used to explore rates of H_2_O_2_ efflux that could reconcile model predictions of Prx3 oxidation states with the experimental observations.

## Author summary

Cancer is a complex disease that caused the deaths of over 9 million people worldwide in 2018, according to the WHO. While great strides have been made in treating many cancers, effective chemotherapies still carry difficult side effects, motivating the search for more targeted and selective treatments that act minimally in healthy cells. The Selective Cancer Killing Hypothesis is based on the idea that some cancers exist at endogenous levels of reactive oxygen species that are higher than healthy cells, so if a patient were systemically treated with a redox-based chemotherapeutic that raises all cells’ levels of reactive oxygen species, only the cancer cells would cross a toxicity threshold. This hypothesis is attractive because it would minimize side effects in healthy cells, but the quantitative knowledge of endogenous oxidant concentrations that would be helpful in refining and testing this hypothesis is not widely established. Our model predicts the range of relevant hydrogen peroxide concentrations in the mitochondria of the HeLa model cancer cell line and suggests experimental measurements of tumor cells and tissues that may be useful in quantifying steady state concentrations of this oxidant.

## Introduction

Reactive oxygen species (ROS) are a class of chemical species that promote diverse phenotypes depending on intracellular concentration, localization and cumulative dose over time, spanning the gamut from homeostasis to toxicity [1,2]. Among ROS, the behavior of hydrogen peroxide (H_2_O_2_) most closely resembles that of a classical signaling molecule, based on the specificity of its reactions and its *in vivo* half-life [3–6]. Mitochondria are putatively a main intracellular source of H_2_O_2_ under basal conditions as a result of the electron transport chain (ETC) and oxidative phosphorylation (OxPhos) [2,7]. This organelle is also hypothesized to be an important site for H_2_O_2_-mediated signaling [8,9].

Previous work in our group has demonstrated that H_2_O_2_ perturbations directed to the mitochondrial matrix elicit a marked toxicity in HeLa cells, especially when contrasted against comparable perturbations delivered in the cytosol [10,11]. This toxicity was both concentration- and time-dependent, indicating the importance of a dynamic understanding of the H_2_O_2_ reaction network. Building upon our experimental results, we sought to further our mechanistic understanding of mitochondrial H_2_O_2_ kinetics by constructing a computational model of the reaction network in this organelle. Detailed molecular mechanisms that connect changes in H_2_O_2_ with phenotypic responses such as changes in mitochondrial morphology, mitochondrial permeability transition (MPT), and programmed cell death have not been elucidated. Since these signaling responses occur during excursions in H_2_O_2_ concentration from the basal steady state, we expect that establishing a quantitative range that can be connected with phenotypic responses will help inform whether particular cysteine residues are likely to become directly oxidized [12]. Existing models on mitochondrial ROS so far have largely fallen into two categories: detailed kinetic models focusing on fast-respiring cells, such as cardiac cells [13,14], or models that exclude the thioredoxin/peroxiredoxin (Trx/Prx) system [15]. Faster rates of cellular respiration [16] and differing abundances of mitochondrial proteins, which have been reported for differing tissue and cell types [17], may lead to differing steady-state H_2_O_2_ concentrations. The Prxs are so abundant and react with H_2_O_2_ with such a high second-order rate constant (10^6^−10^8^ M^-1^s^-1^) that this antioxidant system cannot be neglected [18,19]. Some additional modeling efforts have focused on the kinetics of species other than H_2_O_2_ specifically [20] or on parameter estimation [21]. To our knowledge, this model represents the first kinetic model of the mitochondrial H_2_O_2_ reaction network in a transformed cell line, incorporating the most recent quantitative data specific for HeLa cells.

Here, we implement this model to predict basal H_2_O_2_ concentrations in HeLa cell mitochondria. We also predict network behavior in response to sustained H_2_O_2_ perturbations, including the degree of oxidation of four major antioxidant species present in mitochondria: Prx3, glutathione peroxidase 1 (Gpx1), Prx5, and Gpx4. The mass action kinetics of a network of 30 reactions of 28 chemical species were described using ordinary differential equations and, after parameterization with 30 rate coefficients and species concentrations, solved using MATLAB. Basal mitochondrial H_2_O_2_ as well as reaction network response to H_2_O_2_ perturbations were predicted. Modeling results were compared with experimental data from redox Western blots of the Prxs using the mitochondrially-localized H_2_O_2_ generator D-amino acid oxidase (mito-DAAO). HeLa cells were exposed to a range of D-alanine concentrations, a substrate for mito-DAAO, over time, and Western blots were performed on the cell lysates to observe the change in Prx3 (mitochondrial) and Prx2 (cytosolic) oxidation with the different treatments.

## Methods

### Model formulation: Baseline model

This model was adapted from our previously published kinetic model of the cytosolic antioxidant network, and consists of a system of first-order ordinary differential equations of the form

$$\frac{dC_{i}}{dt}=R_{i}$$ (1)

where *C*_*i*_ is the species concentration, t is time and *R*_*i*_ is the net reaction for that species [22]. It assumes species concentrations are homogeneous throughout the compartment. The baseline model investigates the steady state conditions in the mitochondria, where the only source of endogenous H_2_O_2_ is assumed to be from the ETC due to cellular respiration. For the purposes of this simulation, the rate of H_2_O_2_ generation due to OxPhos is assumed to be invariant.

As a first approximation, transport of H_2_O_2_ between mitochondria and the cytosol is neglected. Release of H_2_O_2_ from isolated mitochondria to the surrounding medium has been measurable, but it has not been possible to measure H_2_O_2_ efflux from mitochondria to the cytosol in intact cells near basal conditions, perhaps due to insufficient sensitivity of existing analytical techniques. By modeling the reaction network with the assumption that H_2_O_2_ efflux is small enough to be neglected and comparing with increasing experimental perturbations to the H_2_O_2_ generation rate, we aim to estimate H_2_O_2_ generation rates for which this assumption breaks down, motivating the need for a refined model that incorporates H_2_O_2_ efflux. The baseline model quickly reaches steady state (less than 1 s), so baseline simulations are carried out to 5 s. The stiff equation solver ode15s in MATLAB was implemented to solve the system of equations.

**Fig 1:**
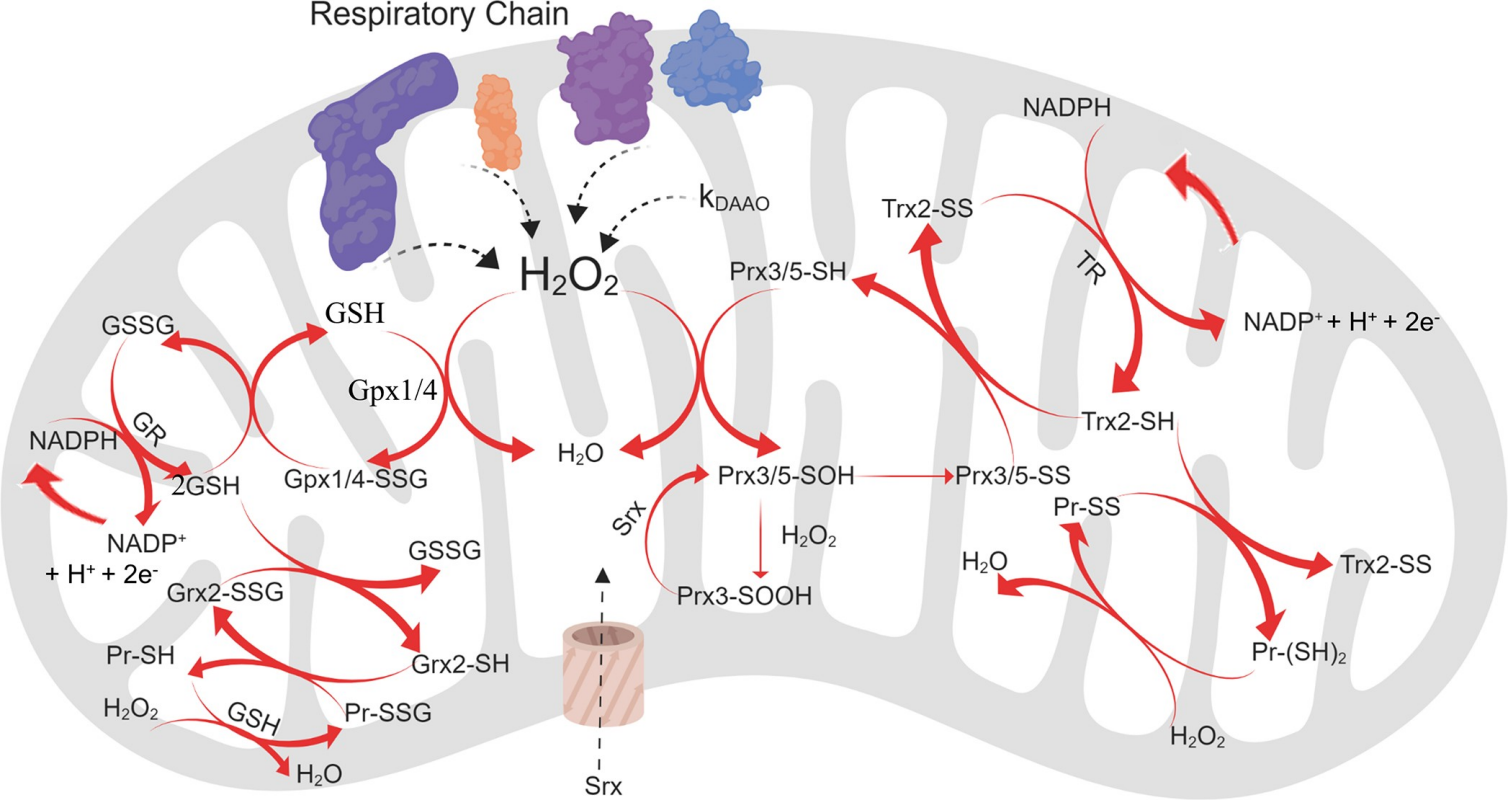
Schematic representation of the H_2_O_2_ reaction network in the mitochondria. H_2_O_2_ is evolved as a result of cellular respiration and the respiratory complexes at a rate that is taken as fixed for the purposes of this model. For the perturbation model only, H_2_O_2_ is also added to the system by a variable source term, *k*_*DAAO*_. It can then participate in reactions with the two Prx isoforms present in the mitochondria (Prx3 and Prx5), which are reduced by Trx2. H_2_O_2_ can also react with either of the Gpx isoforms in the mitochondria (Gpx1 and Gpx4), which involves reduction by GSH. Both of these networks require NADPH for reduction [36,37]. Only Prx3 can undergo the hyperoxidation pathway, forming a sulfinic acid, which is reduced by Srx. Srx is imported into the mitochondria. Finally, H_2_O_2_ can react with protein thiols and dithiols, which are reduced by Trx2 and Grx2, respectively. Image created with Biorender.com.

The main reaction systems that this model captures are the thioredoxin/peroxiredoxin/thioredoxin reductase (Trx/Prx/TR) and the glutathione/glutathione peroxidase/glutaredoxin (GSH/Gpx/Grx) networks. The Prx isoforms found in the mitochondria are Prx3 and Prx5 [23,24], which are reduced by Trx2 [25,26]. Trx2 also reduces disulfide bonds to protein dithiols [27]. Both Gpx1 and Gpx4 are found in the mitochondria, though at low concentrations in HeLa cells [28]. Grx2 is the most abundant mitochondrial Grx isoform, and is responsible for reducing S-glutathionylated proteins [29–31]. While the prior proteins are all considered mitochondrially localized, both GSH and sulfiredoxin (Srx) are generally considered cytosolic molecules that must be imported into the mitochondria [17,32–34]. The mitochondria maintain a large pool of the former, but the latter is only imported based upon a stimulus [33,34]. Catalase is not included because it is not expected to be found in the mitochondria for most cell types, including HeLa cells [17,28,35]. A schematic representation of the reaction networks captured by this model is shown in Fig 1.

The reaction rate parameters for mass action or Michaelis-Menten kinetics in Eq. (1) were found in the peer-reviewed literature or derived from published data. The detailed calculations necessary to derive values of some parameters can be found in S1 Appendix. For any cases where mitochondria-specific values could not be located, the cytosolic equivalent was assumed. These parameters are summarized in Table 1. One difference between previously published models and this one is the treatment of Srx. Previous work [22,38] has assumed zeroth-order kinetics with respect to Srx, leading to the following rate law for hyperoxidized Prx3:

$$\frac{d\left[Prx3-SOOH\right]}{dt}=k_{hyperox}\left[Prx3-SOH\right]\left[H_{2}O_{2}\right]-k_{cat}\left[Prx3-SOOH\right]$$ (2)

where *k*_*hyperox*_ is the rate of hyperoxidation of Prx3 and *k*_*cat*_ is the turnover number reported for Srx by Chang and colleagues [38]. However, overexpression studies have clearly demonstrated an increase in reduction rate of the sulfinic acid with increased Srx concentration [39]. The form of the rate law proposed by Eq (2) fails to capture any dependence on Srx, so we propose a rate law with first-order dependence on Srx as a first approximation:

$$\frac{d\left[Prx3-SOOH\right]}{dt}=k_{hyperox}\left[Prx3-SOH\right]\left[H_{2}O_{2}\right]-k'\;\left[Prx3-SOOH\right]\left[Srx\right]$$ (3)

where *k*' is the estimated second-order rate constant, obtained by dividing 0.18 min^-1^, the first order rate constant reported in [38], by the sulfiredoxin concentration used there. These parameters, *k*_*hyperox*_ and *k’* correspond to *k*_*7*_ and *k*_*8*_ in Table 1, respectively. Glutathione efflux was treated as a first order reaction and the value was determined via trial-and-error to satisfy the constraint that the total glutathione level should not change by more than 5%, just as the total Trx level does not change over the course of the simulations in this work.

**Table 1:** Kinetic parameters. Calculations for parameters that were derived can be found in SI.

Reaction	Parameter
Generation of H_2_O_2_ by OxPhos	*k*_*1*_ = 4 μM/s [16,40]
*k*_*2*_[Gpx1red][H_2_O_2_]	*k*_*2*_ = 60 μM^-1^s^-1^ [35,41]
*k*_*3*_[Gpx1ox][GSH]	*k*_*3*_ = 0.04 μM^-1^s^-1^ [42]
*k*_*4*_[Gpx-SSG][GSH]	*k*_*4*_ = 10 μM^-1^s^-1^ [42]
*k*_*20*_[NADP^+^]/(k_5_+[NADP^+^])	k_5_ = 57 μM [43]
*k*_*6*_[Prx3-SH][H_2_O_2_]	*k*_*6*_ = 20 μM^-1^s^-1^ [23]
*k*_*7*_[Prx3-SOH][H_2_O_2_]	*k*_*7*_ = 0.014 μM^-1^s^-1^ [44]
*k*_*8*_[Prx3-SOOH][Srx]	*k*_*8*_ = 3x10^-3^ μM^-1^s^-1^ [38]
*k*_*9*_[Prx3-SOH]	*k*_*9*_ = 20 s^-1^ [44]
*k*_*10*_[Prx3-SS][Trx2-SH]	*k*_*10*_ = 0.22 μM^-1^s^-1^ [26,45]
*k*_*11*_[GSH]	*k*_*11*_ = 7.4x10^-5^ s^-1^ [46]
*k*_*12*_[Pr-SH][H_2_O_2_]	*k*_*12*_ = 1x10^-4^ μM^-1^s^-1^ [47,48]
*k*_*13*_[Pr-SOH][GSH]	*k*_*13*_ = 0.12 μM^-1^s^-1^ [49,50]
*k*_*14*_[Grx2-SH][Pr-SSG]	*k*_*14*_ = 0.01 μM^-1^s^-1^ [51]
*k*_*15*_[Grx2-SSG][GSH]	*k*_*15*_ = 0.04 μM^-1^s^-1^ [52]
*k*_*16*_[Pr-(SH)_2_][H_2_O_2_]	*k*_*16*_ = 1x10^-4^ μM^-1^s^-1^ [47]
*k*_*17*_[Pr-SS][Trx2-SH]	*k*_*17*_ = 1x10^-4^ μM^-1^s^-1^ [47]
*k*_*18*_[GSSG][NADPH]	*k*_*18*_ = 3.2 μM^-1^s^-1^ [53]
*k*_*19*_[Trx2-SS][NADPH]	*k*_*19*_ = 20 μM^-1^s^-1^ [54]
*k*_*20*_[NADP^+^]/(k_5_+[NADP^+^])	*k*_*20*_ = 375 μM/s [43]
GSH import	*k*_*21*_ = 0.48 μM/s [55]
GSH efflux k_22_[GSH]	*k*_*22*_ = 9.6×10^−5^ s^-1^ described in text
k_23_[Prx5-SH][H_2_O_2_]	*k*_*23*_ = 0.3 μM^-1^s^-1^ [23,25]
k_24_[Prx5-SOH]	*k*_*24*_ = 14.7 s^-1^ [25]
k_25_[Prx5-SS][Trx2-SH]	*k*_*25*_ = 2 μM^-1^s^-1^ [25]
k_26_[Gpx4red][H_2_O_2_]	*k*_*26*_ = 0.05 μM^-1^s^-1^ [56]
k_27_[Gpx4ox][GSH]	*k*_*27*_ = 0.02 μM^-1^s^-1^ [56]
Generation of H_2_O_2_ by DAAO	*k*_*28*_ = *k*_*DAAO*_ described in text
Srx import	*k*_*29*_ = 1.23x10^-5^ μM/s [57]
H_2_O_2_ efflux	*k*_*30*_ = *k*_*efflux*_ described in text

Species abundances for model initialization were either found in literature, calculated from published datasets, or calculated based on molar balances and rate laws. Species that were found in literature or calculated using published datasets are summarized in Table 2, and species that were derived from molar balances and rate laws are summarized in Table 3. Prx3-SH abundance is given as a range rather than a single value. This is the result of the calculations that are necessary to convert per-cell protein copy numbers from the proteomics dataset in [28] to a per mitochondrion concentration. For these calculations, mitochondrial volume was taken as 0.29 μm^3^ [58] and mitochondrial number in a HeLa cell has been reported to range from 383–882 [59]. A total protein density throughout the cell was reported as 2x10^5^ mg/L in [28] so we assumed this density was invariant between organelles. Additional details regarding these calculations can be found in S1 Appendix.

**Table 2:** Initial species abundances.

Species	Concentration (μM)
Prx3-SH	48–110 [28,58,59]
Prx5-SH	14 [28,58,59]
Gpx1	1.5x10^-2^ [17,28,58,59]
Gpx4	0.23 [17,28,58,59]
Grx2	1 [28,58,59]
Trx2-SH	7.7 [28,58,59,61,62]
Trx2-SS	0.075 [63]
GSH	5x10^3^ [23]
GSSG	1.78 [63]
NADPH	30 [64]
NADP^+^	0.03 [65]
Pr-SH	1x10^-3^ [47]
Pr-(SH)_2_	1.09x10^3^ [63]
Srx	8.8x10^-3^ [28]

**Table 3:** Derived initial species abundances.

Species	Concentration (μM)
H_2_O_2_	2x10^-3^ – 4x10^-3^
Prx3-SOH	0.18–0.20
Prx3-SS	2.2–2.3
Prx3-SOOH	0.19–0.42
Prx5-SOH	5x10^-4^ – 1x10^-3^
Prx5-SS	5x10^-4^ – 1x10^-3^
Gpx1ox	8x10-6 – 2x10^-5^
Gpx1-SSG	3x10-8 – 7x10^-8^
Gpx4ox	0
Gpx4-SSG	0
Grx2-SSG	1x10^-16^
Pr-SOH	3x10-13 – 6x10^-13^
Pr-SSG	1x10-8 – 3x10^-8^
Pr-SS	0.26–0.59

While all the proteins that were calculated based on the data in [28] produced a range of possible values depending on the number of mitochondria per cell, Prx3-SH was by far the most abundant and has a very high rate constant for reaction with H_2_O_2_. Therefore, we considered the range of Prx3-SH concentrations explicitly while taking the median value for other protein concentrations calculated from [28]. Notably, the abundances of Gpx1 and Gpx4 listed in Table 2, calculated from the proteomics dataset in [28], are much lower than values suggested in previous work with hepatocytes (2 and 1 order of magnitude lower, respectively) [60]. Because several species are initialized by molar balance, the range in Prx3-SH initialization results in several species in Table 3 to initialize differently depending on its concentration.

### Quantifying uncertainty in model predictions

#### Monte Carlo parameter sampling

Based on the feasible concentration range of Prx3-SH in HeLa mitochondria, we generated 10,000 random samples (shown in S1 Appendix) spread uniformly throughout the feasible space using the following equation [66]:

$$C_{\mathrm{Prx}3‐\mathrm{SH}}=U\left(0,1\right)\times \left[C_{\mathrm{Prx}3‐\mathrm{SH}}^{\max}-C_{\mathrm{Prx}3‐\mathrm{SH}}^{\min}\right]+C_{\mathrm{Prx}3‐\mathrm{SH}}^{\min}$$ (4)

Here, U(0,1) refers to a single uniformly distributed random number in the range of 0 to 1, and $C_{Prx3‐SH}^{min}$ is 48 μM and $C_{Prx3‐SH}^{max}$ is 110 μM. A set of randomly generated Prx3-SH concentrations was used as initial conditions for implementation of ODE simulations, providing a distribution of predicted steady-state concentrations of each species of interest.

#### Sensitivity analysis

In order to calculate the sensitivity of predicted steady-state H_2_O_2_ concentrations and protein redox balances to the values of model parameters used, the finite difference approximation method was used [67]. The sensitivities were calculated using the following equation:

$$s_{i}\left(t\right)=\frac{\partial C_{j}\left(t\right)}{\partial k_{i}}=\frac{C_{j}\left(k_{i}+\Delta k_{i},t\right)-C_{j}\left(k_{i},t\right)}{\Delta k_{i}}$$ (5)

where *s*_*i*_ is the sensitivity corresponding to parameter *k*_*i*_ and *C*_*j*_ is the concentration of the species of interest (e.g. H_2_O_2_ or Prx3-SS). Parameters were perturbed by 10% to reflect an estimate of typical experimental error, and sensitivities were normalized to adjust for differences in orders of magnitude:

$$\bar{s_{i}}\left(t\right)=\frac{\partial C_{j}\left(t\right)/C_{j}\left(t\right)}{\partial k_{i}/k_{i}}$$ (6)

Sensitivities of the basal, steady-state model predictions were calculated at 5 s, and here, we report only $\bar{s_{i}}$.

### Model formulation: H_2_O_2_ perturbation

The second part of this modeling endeavor sought to investigate the effects of a source of H_2_O_2_ perturbation, similar to what is introduced by the synthetic biology tool D-amino acid oxidase (DAAO) targeted to the mitochondrial matrix. This was modeled as a constant source term, *k*_*DAAO*_, within the H_2_O_2_ rate equation, as depicted in Fig 1. Because we were interested in how the network would respond to perturbations of varying magnitudes, we swept this parameter across a range of values until we reached an upper limit of possible physiological relevance, which we defined as the complete collapse of the Prx3 system. This part of the simulation was carried out to 3600 s (1 hr).

### Comparison of model predictions with experimental data

#### Cell culture

HeLa cells that had previously been transfected by lentivirus to stably express a mitochondrially-targeted D-amino acid oxidase (mito-DAAO) H_2_O_2_ generator [10] were maintained in Dulbecco’s modified Eagle’s medium (DMEM; Lonza), supplemented with 10% fetal bovine serum (FBS; ATCC) at 37°C in a humidified atmosphere with 5% CO_2_. Cells were passaged approximately every 3 days and were maintained under selective pressure using 6 μg/mL puromycin (Sigma) until 24 hrs before any experiments.

#### Analysis of Prx response to mitochondrial H_2_O_2_ perturbations

HeLa cells expressing mito-DAAO were seeded at 3.5x10^5^ cells/well in 6-well plates ~18 hours prior to the start of generation (target confluence ~50% at start of experiment). Cells were exposed to 5 μM flavin adenine dinucleotide (FAD; Sigma) and concentrations of D-alanine (Sigma) from 0–25 mM in Roswell Park Memorial Institute 1640 medium (RPMI; Invitrogen) without phenol red. At the end of the H_2_O_2_ generation period, cells were washed with ice cold 1x phosphate buffered saline (PBS) and then incubated on ice with 2 mL 100 mM methyl methanethiosulfonate (MMTS; Sigma) for 30 min to block free thiols. Cells were washed twice more with cold PBS, then lysed in 100 μL of lysis buffer (0.5% Triton X-100 (Sigma), 1x HALT protease and phosphatase inhibitor (ThermoFisher), 1x PBS). Lysates were centrifuged on a cooled rotor for 10 min at 10,000xg and the supernatant was collected and stored at -80°C for further analysis. Western blotting was carried out according to the protocol in [68]. Proteins were separated by non-reducing SDS-PAGE using a pre-cast 12% polyacrylamide stain-free gel (Bio-Rad). Following SDS-PAGE, the gel was activated for 45 s using a ChemiDoc MP (Bio-Rad), then proteins were transferred to a polyvinylidene difluoride (PVDF) membrane for immunoblotting. Blots were blocked using Odyssey blocking buffer (Licor), and incubated with primary antibodies against Prx3 (Abcam, ab73349), Prx2 (R&D Systems, AF3489), and Hsp60 (R&D Systems, Clone# 264233) either overnight at 4°C or 2 hr at room temperature. Endogenous Hsp60 was used to account for differences in loading. Blots were incubated for 1 hr at room temperature with Licor IRDye secondary antibodies. The ChemiDoc MP system was used to image the blots, then ImageJ was used to quantify the images for densitometry.

### Statistical analysis

Analysis of variance (ANOVA) was used to test for trends in the fractional oxidation of the Prx protein, as measured by Western blots. At least three biological replicates per time point were used for trend testing. Post-hoc Tukey’s Honest Significant Difference (Tukey-HSD) testing was performed to determine which sample means were different from the control (0 mM D-ala) within each time point.

### Model refinement: H_2_O_2_ perturbation

An H_2_O_2_ efflux reaction was added to represent transport of H_2_O_2_ out of the mitochondria and into the cytosol. Monte Carlo parameter sampling using sets of 10,000 random sample points for *k*_*DAAO*_ and *k*_*efflux*_ were generated in uncertain ranges of these parameters. For high *k*_*DAAO*_ values where efflux may be important, the minimum and maximum of *k*_*DAAO*_, or $\left(k_{DAAO}^{min},k_{DAAO}^{max}\right)$, were set to (50, 100), where the units are μM/s. Two efflux cases were considered, termed low and high. For low *k*_*efflux*_, $\left(k_{efflux}^{min},k_{efflux}^{max}\right)$ was set to (0, 50), and for high *k*_*efflux*_, $\left(k_{efflux}^{min},k_{efflux}^{max}\right)$ was set to (50, 100), where the units are again μM/s. Equations following the form of (4) above together with uniformly distributed random numbers in the range of 0 to 1 were used to produce sets of parameter values, further described in S1 Appendix, that were used in implementation of ODE simulations.

## Results

**Fig 2:**
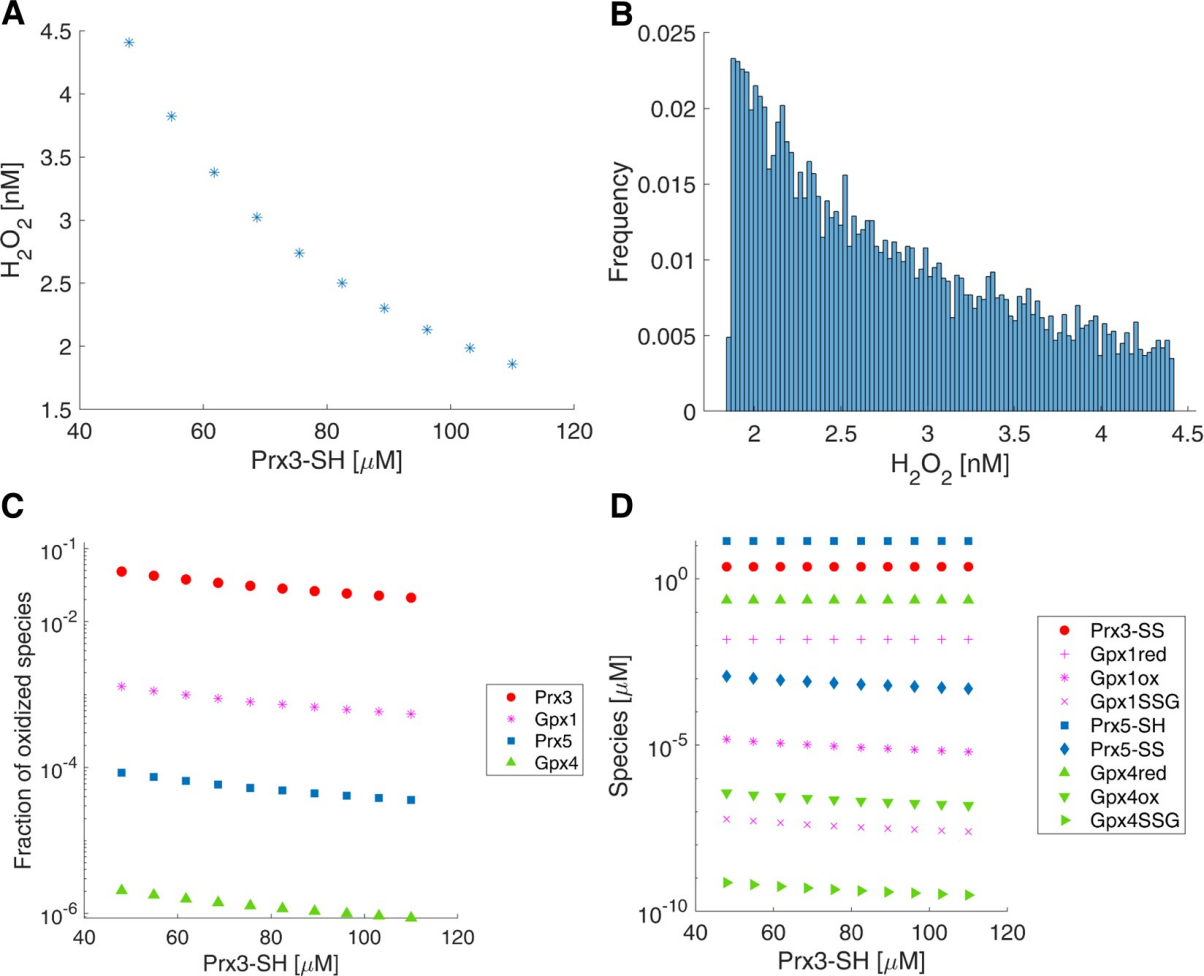
Baseline conditions. A) Steady state, basal [H_2_O_2_] in a mitochondrion with fixed H_2_O_2_ generation from OxPhos (4 μM/s) for different initial pools of Prx3-SH, based on possible range calculated from [28] and [60]. B) Distribution of basal [H_2_O_2_] based on simulation results with 10,000 randomly sampled initial Prx3-SH pools in the same range examined in A). C) Fraction of dimerized Prx3 (Prx3-SS/total Prx3), dimerized Prx5, and oxidized GPx species versus initial concentration of Prx3-SH for fixed H_2_O_2_ generation from OxPhos (4 μM/s). D) Steady state concentrations of oxidized and reduced forms of the four major antioxidants in the mitochondria as a function of initial Prx3-SH concentration with fixed H_2_O_2_ generation from OxPhos (4 μM/s). Similar plots for a fixed H_2_O_2_ generation rate from OxPhos of 11 μM/s can be found in S1–S3 Figs.

The first quantity investigated was the basal, steady-state concentration of H_2_O_2_ in mitochondria, which was predicted to range between 1.8–4.4 nM (Fig 2A). This steady-state concentration showed a strong inverse dependence on the concentration of Prx3 within a mitochondrion. Fig 2A shows that a two-fold increase in Prx3 concentration leads to a two-fold decrease in steady state H_2_O_2_ concentration. The range of Prx3 concentrations examined in Fig 2A reflects the current state of knowledge of this mitochondrial protein’s concentration. Copy numbers of Prx3 proteins per cell have been calculated from pooled lysates of many cells [28], and number of mitochondria per cell have been measured [60], narrowing the likely range of Prx3 concentrations per mitochondrion, and thus basal, steady state H_2_O_2_ concentrations within mitochondria, to the ranges that are plotted in 2A. To supplement the ten single-point calculations presented in Fig 2A, Monte Carlo parameter sampling within the same range of Prx3 concentrations was used to further investigate the range of steady-state mitochondrial H_2_O_2_ concentrations, resulting in the distribution shown in Fig 2B.

We next investigated the effect of the total available pool of Prx3 on the dimer fraction of Prx3, calculated as

$$\mathrm{fraction}\;\mathrm{dimerized}=\frac{Prx3SS}{Prx3_{total}}$$ (7)

a quantity is often measured experimentally by Western blotting, and at baseline can characterize the variability between different cell types [10,69]. Fig 2C plots this quantity as well as the fractional oxidation of other peroxidases found within the mitochondria. Similar to the basal, steady-state H_2_O_2_ concentration, the fractions of oxidized peroxidases all demonstrate an inverse relationship with the total pool of Prx3. Only Prx3 experiences any significant degree of oxidation at baseline, as shown in Fig 2C and 2D and summarized in Table 4. The fractional oxidation in Table 4 represents the dimer fraction for the Prxs and the fraction of Gpxox + GpxSSG for the Gpxs. The basal model predicts that only 2 to 5% of the total Prx3 pool is engaged in maintaining H_2_O_2_ at low nM concentrations, leaving a large excess of Prx3-SH.

**Table 4:** Summary of fractional oxidation of major antioxidants in the network.

Species	Fraction oxidized
Prx3 (dimer)	0.021–0.048
Gpx1	5.4x10-4–1.3x10^-3^
Prx5 (dimer)	3.6x10-5–8.5x10^-5^
Gpx4	8.7x10-7–2.1x10^-6^

**Fig 3:**
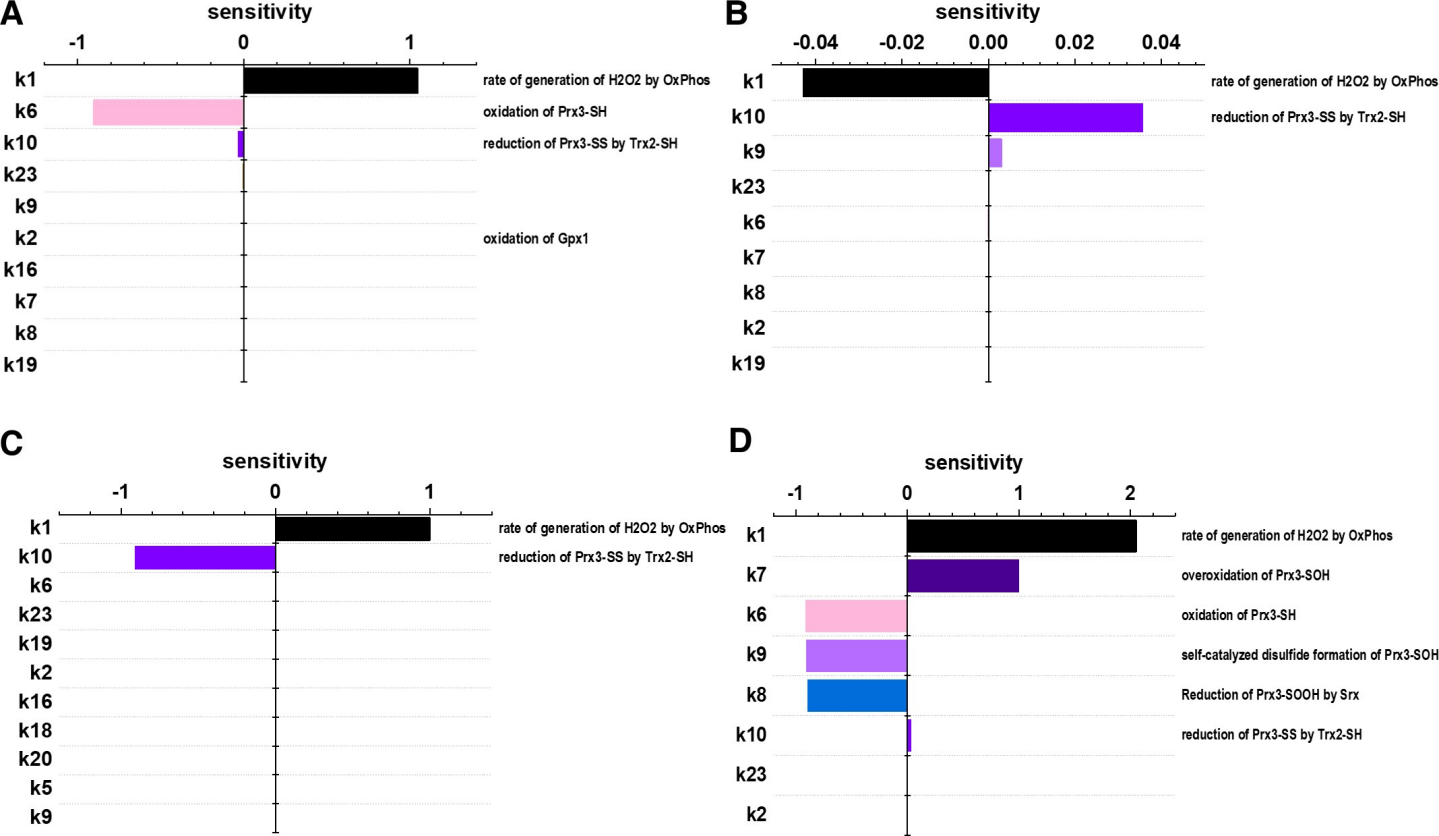
Sensitivity analysis to identify dominant H_2_O_2_ clearance reactions and assess the impact of uncertainty in parameter values on predicted steady-state basal concentrations. Tornado plots of sensitivities to model rate parameters for A) [H_2_O_2_], B) [Prx3-SH], C) [Prx3-SS], and D) [Prx3-SOOH] when *k*_*DAAO*_ = 0. Plots show model rate constants in descending order of sensitivities (absolute value) and are truncated to show only sensitivities above |10^−5^|.

In order to evaluate the impact that parameter uncertainties may have on the model predictions, we performed a sensitivity analysis. The value of $\bar{s_{i}}$ can inform us about both the magnitude and direction that changes in a particular parameter will have on the predictions for a given species of interest. For example, a sensitivity of 1 indicates that a 10% increase in the parameter resulted in a 10% increase in the model output, and likewise, a sensitivity of -1 would signify a 10% decrease in the model output. Fig 3 depicts tornado plots of the sensitivities of the predicted basal steady-state concentrations of H_2_O_2_ (A), Prx3-SH (B), Prx3-SS (C), and Prx3-SOOH (D) to kinetic parameters within the model. These plots order the parameters from greatest to least effect on the model output. The model prediction for [H_2_O_2_] was most sensitive to *k*_*1*_, the rate of generation of H_2_O_2_ by OxPhos, closely followed by the rate constant of oxidation of Prx3-SH, *k*_*6*_. Prx3-SS was similarly sensitive to *k*_*1*_ and was also sensitive to *k*_*10*_, the rate constant of reduction of Prx3-SS by Trx2-SH. Prx3-SH was not very sensitive to any single model parameter, and Prx3-SOOH was sensitive to several parameters, especially *k*_*1*_. *k*_*1*_ appeared in all four sensitivity analyses as a top parameter, indicating its importance to all the model predictions. The sensitivity analysis, therefore, pointed to the model’s overall dependence on the rate of H_2_O_2_ input into the system and the kinetic parameters within the Trx2/Prx3 pathway.

**Fig 4:**
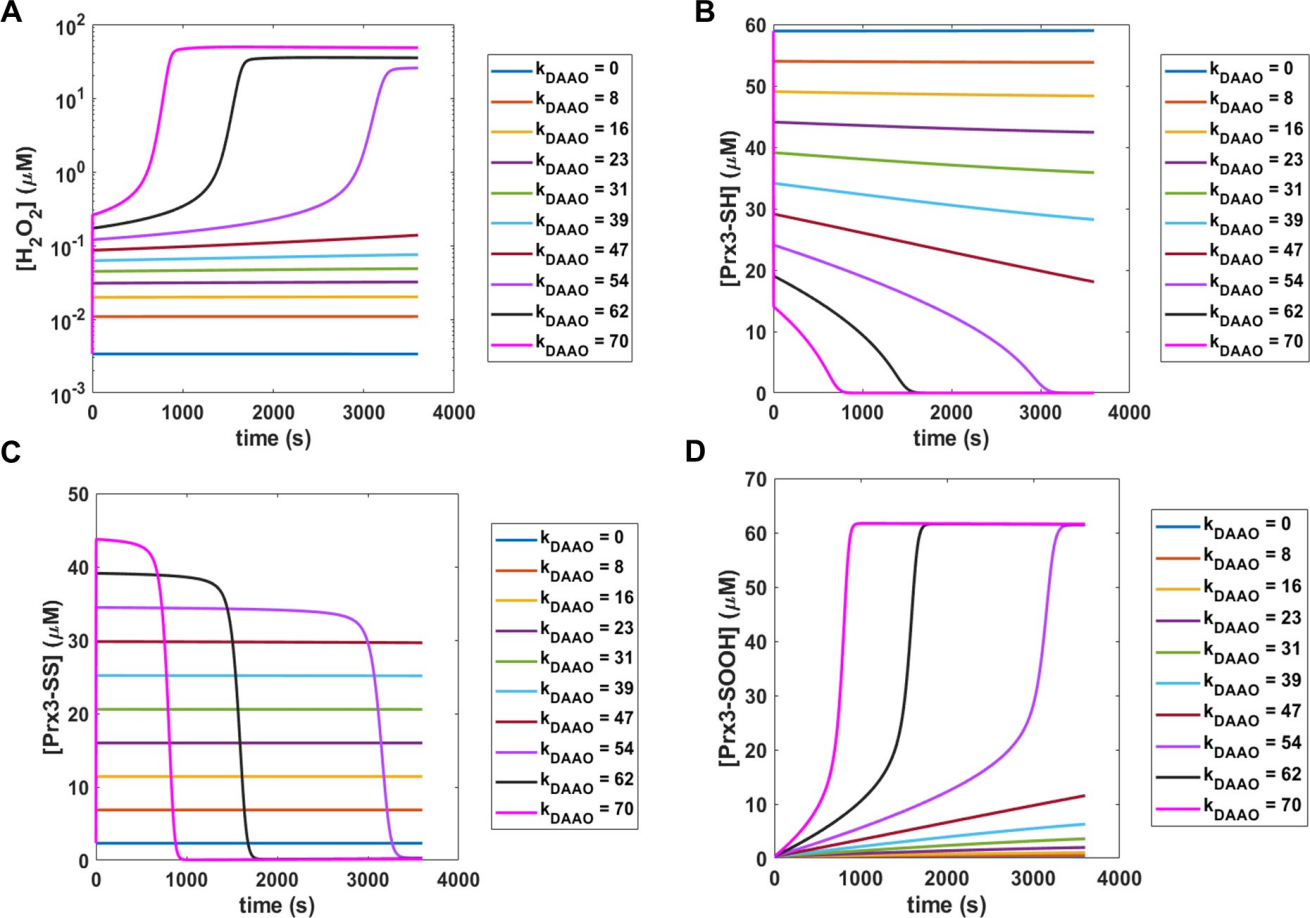
Perturbation analysis. Plots showing the dynamics of A) [H_2_O_2_], B) [Prx3-SH], C) [Prx3-SS], and D) [Prx3-SOOH] with an initial Prx3-SH concentration of 62 μM for increasing values of *k*_*DAAO*_. All values of *k*_*DAAO*_ in μM/s.

Once the baseline was established, we next sought to evaluate the network response to H_2_O_2_ perturbations. To clearly show dynamic behavior during a range of perturbations, we fixed the initial concentration of Prx3-SH at 62 μM, one of the ten concentrations examined in Fig 2 and a value that is close to a previous experimental measurement [35]. The magnitude of the perturbation term, *k*_*DAAO*_, was varied up to 70 μM/s, at which point the reaction network showed evidence of nearing saturation, demonstrated in Fig 4 by changes in the shape of the H_2_O_2_ traces at high perturbation rates. Fig 4A depicts the concentration of H_2_O_2_ over time for each perturbation rate simulated. For all but the highest values of *k*_*DAAO*_, the H_2_O_2_ concentration settled out to a new steady state within milliseconds. It can be observed from the plots of Prx3 dynamics in Fig 4B–4D that this antioxidant pool controls the dynamics of mitochondrial H_2_O_2_. Prx3-SH concentration reached a new steady state for each perturbation rate, reflecting the predicted H_2_O_2_ behavior. Prx3-SS concentrations jumped to correspondingly higher levels for each perturbation rate, and in the range of 23–47 μM/s of increased H_2_O_2_ generation, slowly declined over the 1 hr simulation, accompanied by a slow increase in the Prx3-SOOH isoform. At perturbations above 47 μM/s, the H_2_O_2_ concentration followed a sigmoid trend that reached an asymptote of tens of μM. This was accompanied by a collapse of the Prx3 network, demonstrated by Fig 4B and 4C. At these very high perturbations, all of the Prx3 became trapped as the hyperoxidized isoform, shown in Fig 4D. It is only when the capacity of the Prx3/Trx/TR system was exceeded that other antioxidants were able to kinetically compete and react with H_2_O_2_, as summarized by Table 5, which lists the fractional oxidation for the four major antioxidant species at each perturbation rate, following the same convention as in Table 4. It is important to note that the predicted steady states that result from this H_2_O_2_ perturbation analysis are the net effect of the rate of H_2_O_2_ generation by OxPhos and the additional *k*_*DAAO*_ generation term; a higher OxPhos generation rate would result in a lower *k*_*DAAO*_ needed to cause collapse of the Prx3 system.

**Table 5:** Summary of fractional oxidation with changing H_2_O_2_ perturbations for the major antioxidants in the network at 1 hr with an initial Prx3-SH concentration of 62 μM.

*k*_*DAAO*_ (μM/s)	Fraction Oxidized			
	Prx3 (SS dimer)	Gpx1	Prx5 (SS dimer)	Gpx4
0	0.04	7.68x10^-4^	6.52x10^-5^	1.23x10^-6^
8	0.11	2.47x10^-3^	2.11x10^-4^	3.96x10^-6^
16	0.18	4.56x10^-3^	3.90x10^-4^	7.31x10^-6^
23	0.26	0.01	6.21x10^-4^	1.16x10^-5^
31	0.33	0.01	9.44x10^-4^	1.77x10^-5^
39	0.41	0.02	1.47x10^-3^	2.74x10^-5^
47	0.48	0.03	2.69x10^-3^	5.02x10^-5^
54	0.01	0.85	0.25	0.01
62	3.83x10^-3^	0.89	0.29	0.01
70	2.82x10^-3^	0.92	0.33	0.02

**Fig 5:**
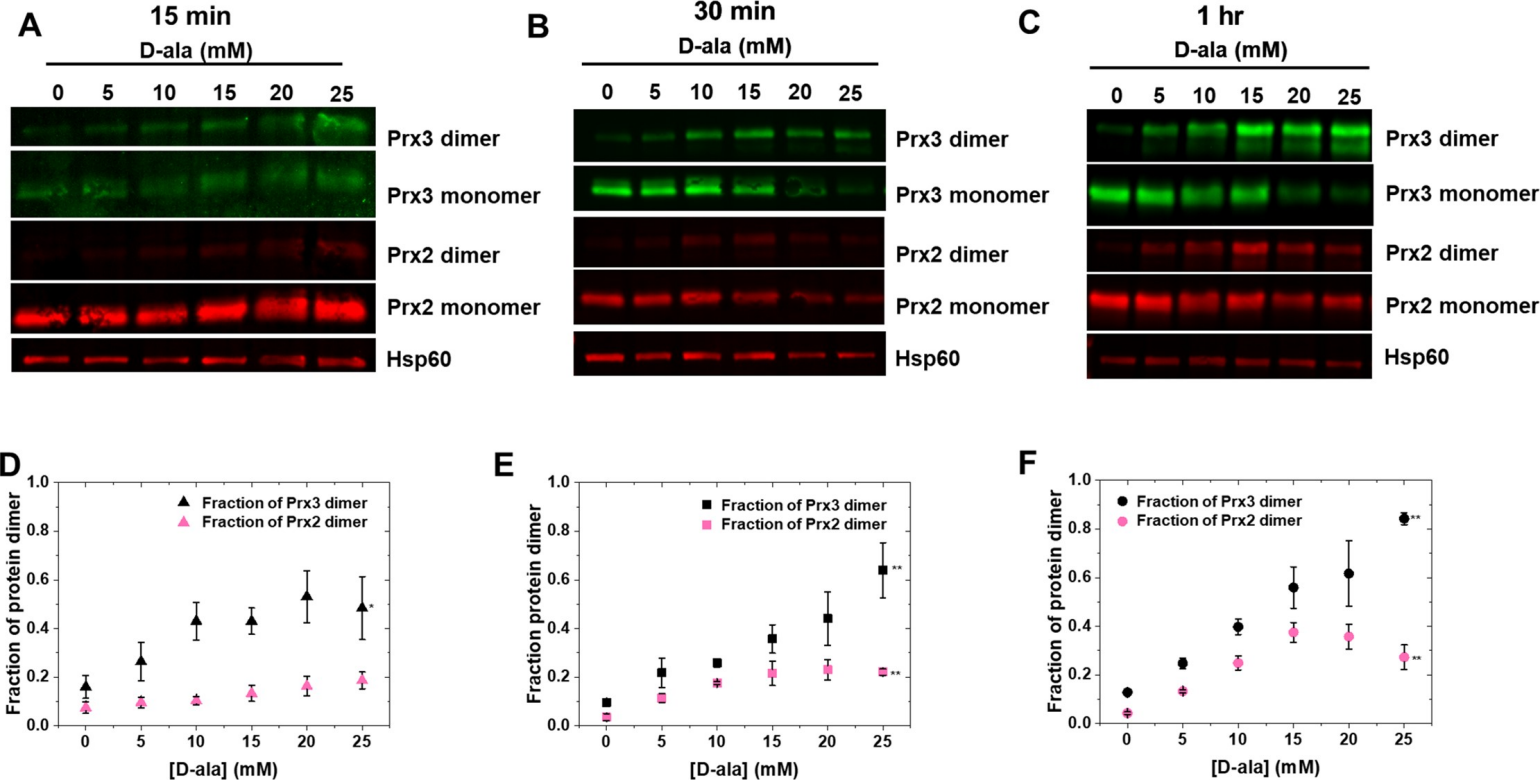
Comparison with experimental data. Western blot analysis of the oxidized (dimer) and reduced (monomer) Prx isoforms after A) 15 min, B) 30 min, and C) 1 hr of H_2_O_2_ generation by mito-DAAO, with corresponding densitometry plots in D, E, and F, respectively. * represents P<0.05 for one-factor ANOVA, ** represents P<0.01 for one-factor ANOVA. Full Western blot images can be found in S5–S13 Figs.

In order to experimentally investigate the trends predicted by the model, we used the genetically-encoded H_2_O_2_ generator mito-DAAO, which localizes a H_2_O_2_ perturbation to the mitochondrial matrix [10,70]. We varied the concentration of D-alanine (D-ala) substrate the cells were exposed to for up to 1 hr, then probed the Prx3 and Prx2 isoforms using redox Western blots. Prx2 is found in the cytosol and provided a means to assess H_2_O_2_ efflux from the mitochondria to the cytosol. The Western blot results are summarized in Fig 5. The experimental data demonstrates consistently higher fractions of oxidized, dimer Prx3 than the model predicts, and this discrepancy is most prominent at high perturbations. Where the model predicts a maximum fractional oxidation of Prx3 to the disulfide-linked dimer form of around 0.5, the experimental data continues to rise monotonically, reaching a fraction of oxidized Prx3 as high as 0.8. Thus, the model over-predicts hyperoxidation. An increase in the concentration of Prx3 during the perturbation could contribute to a lesser degree of oxidation than expected. We examined whether the total amount of Prx3 increased during increased H_2_O_2_ generation for up to 1 hour, and found no evidence of increases in total Prx3 abundance (S4 Fig).

The Prx2 data demonstrate increased H_2_O_2_ flux in the cytosol at certain perturbations after 15 min. At 15 min, while one-way ANOVA testing determined there was a statistically significant trend in Prx3 mean fractional oxidation at the 95% confidence level (P = 0.041), the same test found the Prx2 means to *not* differ across D-ala concentrations (P = 0.095) suggesting an undetectable amount of transport at this time scale. However, at subsequent times, both Prx3 and Prx2 oxidation demonstrated significant trends at the 99% confidence level, as determined by one-way ANOVA (P = 0.004 and P = 0.003 for Prx3 and Prx2 at 30 min, P = 1.76 x 10^−5^ and P = 2.05 x 10^−5^ for Prx3 and Prx2 at 1 hr). This suggests that transport effects may be playing a larger role at these longer times, as Prx2 oxidation becomes increasingly significant. This efflux of H_2_O_2_ from the mitochondria to the cytosol may explain, at least in part, why Prx3 did not collapse into the hyperoxidized form after reaching dimer fractions above 0.5 as predicted by a model that neglects H_2_O_2_ efflux.

**Fig 6:**
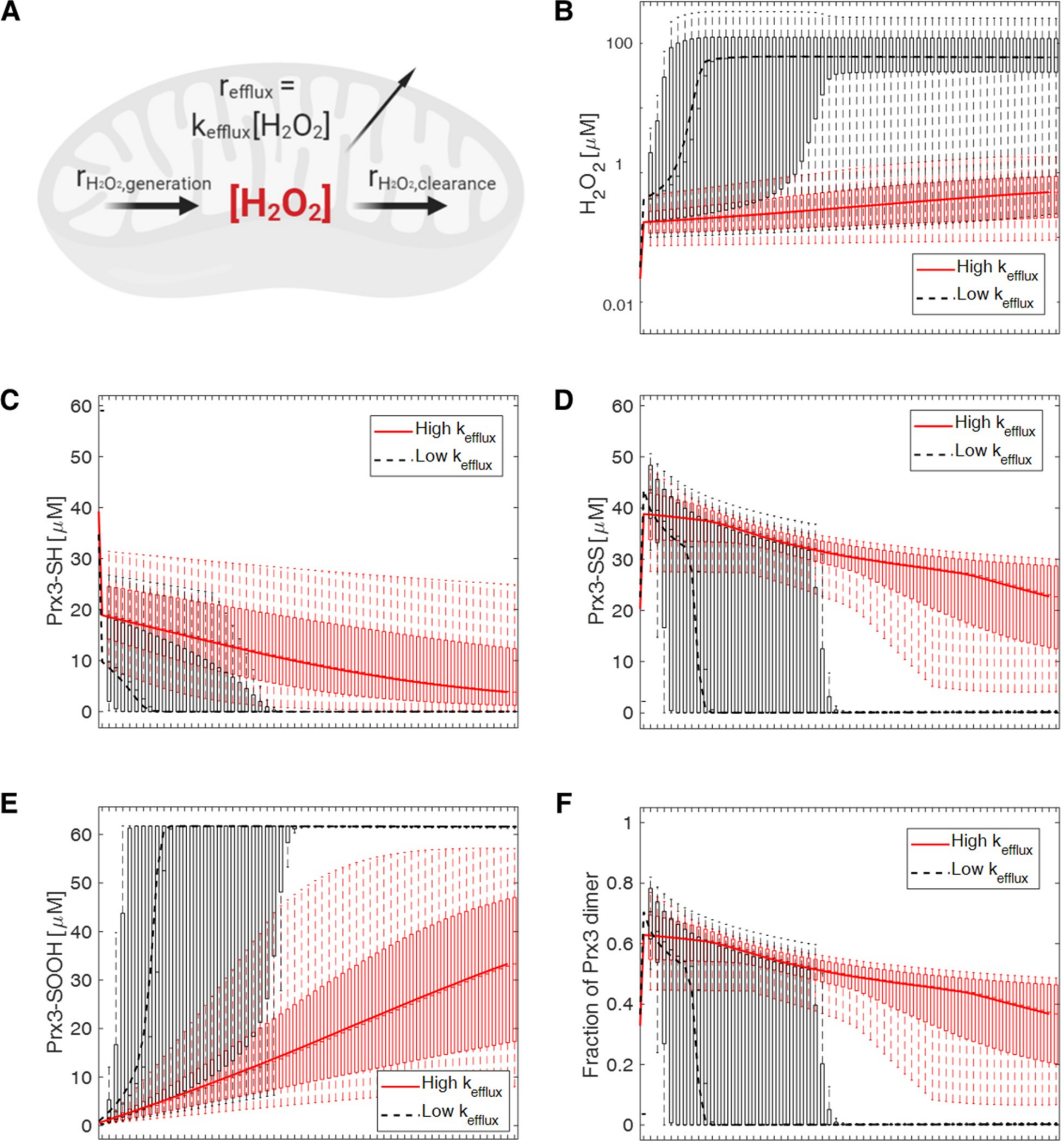
Model refinement and predictions with H_2_O_2_ efflux rate. A) Schematic of a refined kinetic model that includes the rate of H_2_O_2_ efflux. B) Model predictions of H_2_O_2_ dynamics under high production rates of H_2_O_2_ with either low or high efflux rate. 10,000 samples were randomly generated based on uniform distribution of rates ranging from 50 to 100 μM/s for *k*_*DAAO*_, 0 to 50 μM/s for low *k*_*efflux*_ conditions, and 50 to 100 μM/s for high *k*_*efflux*_ conditions. Lines represent median values. Points represented in box plot form show 2.5, 25, 75, and 97.5 percentile values at times ranging from 0 to 60 minutes. Dynamic behaviors of Prx3-SH, Prx3-SS, Prx3-SOOH, and the fraction of Prx3 dimer are presented in C), D), E) and F).

Motivated by this experimental evidence, we refined the kinetic model to include an H_2_O_2_ efflux reaction that is dependent on the concentration of H_2_O_2_ as shown in Fig 6A. The range of values of *k*_*DAAO*_ where H_2_O_2_ efflux is substantial and the values of *k*_*efflux*_ are both uncertain. Monte Carlo parameter sampling was used to explore ranges of both of these variables. Fig 6 shows the results of sampling high values of *k*_*DAAO*_ and comparing two cases: a range of low values of *k*_*efflux*_ and a range of high values of *k*_*efflux*_.

When the range of *k*_*efflux*_ values is lower than the range of high *k*_*DAAO*_ values, including those predicted in Fig 4 to result in collapse of the Prx3 system, Fig 6B shows system behavior that is similar to Fig 4. The concentration of H_2_O_2_ in mitochondria increases dramatically into the micromolar range as Prx3 becomes completely hyperoxidized (Prx3-SOOH) on the minutes timescale. Sampling *k*_*efflux*_ values in the same range as the large *k*_*DAAO*_ values that were sampled results in dynamics of the system that are more similar to, though not the same as, the experimental data (S14 Fig). The high *k*_*efflux*_ cases in Fig 6B–6F represent a range of values that prevent collapse of the Prx3 system, maintaining H_2_O_2_ concentrations in the nanomolar range and resulting in dimer fractions that are larger than 0.4. This modelling approach still seems to over-predict hyperoxidation.

## Discussion

Our analysis of mitochondrial H_2_O_2_ metabolism found that Prx3 is the antioxidant in the mitochondrial H_2_O_2_ reaction network that controls the steady state concentration of H_2_O_2_, as has been previously hypothesized [35]. In HeLa cells, we predicted basal H_2_O_2_ to be in the range of 2–4 nM. Further, we examined the impact of increasing H_2_O_2_ generation rates on the reaction network. Because of the reducing capacity of Prx3, the mitochondrial reaction network is able to control H_2_O_2_ perturbations in the low μM/s range without participation from Gpx1, Prx5, and Gpx4. Only at perturbations that cause total saturation of the Prx3 system do we expect oxidation of Gpx1, Prx5, and Gpx4. Thus, under most circumstances, Prx5 and Gpx4 are not expected to react directly with H_2_O_2_, consistent with the peer-reviewed literature describing their other roles. It has been previously reported that, though Prx5 and Gpx4 are able to react with H_2_O_2_, that is not their primary biological function; Prx5 is the putative reductant of reactive nitrogen species and Gpx4 is hypothesized to react with lipid hydroperoxides [23,56,71].

A model that neglects efflux of H_2_O_2_ from the mitochondria to the cytosol predicts a great deal of hyperoxidation of Prx3 at moderate to large perturbation rates. This is inconsistent with experimental observations of monotonically increasing dimeric Prx3-SS in redox Western blots as a function of increasing H_2_O_2_ generation rates (Fig 5). If hyperoxidation of Prx3 became prevalent at a particular increased H_2_O_2_ generation rate, it would be evidenced experimentally by a decrease in the intensity of the dimeric Prx3-SS band. This behavior was observed for Prx2 (Fig 5F). Prx3 is known to be less prone to hyperoxidation as compared with Prx2, as it has faster resolution kinetics of disulfide formation [44,72]. One limitation to accurately predicting Prx3 hyperoxidation is that the reduction kinetics of the sulfinic acid form of Prx3 have not been well characterized, nor the dynamics of Srx import into and export from the mitochondria. In addition, the reduction of Srx itself is still poorly understood [73]. More careful quantitative analyses of the kinetics governing this reaction pathway will improve our understanding of the dynamics of hyperoxidation. However, the largest contributor to the inconsistency of the model’s predictions with experimental data at large perturbation rates arises from neglecting efflux. Redox Western blots of mitochondrial and cytosolic Prx isoforms showed that while efflux of H_2_O_2_ from the mitochondria to the cytosol wasn’t detectable at 15 minutes, it became increasing important at longer times over the range of perturbations studied.

Physiologically, a variety of interesting reactions within and across the mitochondrial membranes may occur over the range of perturbation rates we studied, including aquaporin or other pore-mediated diffusion of H_2_O_2_ into the cytosol and even possible depolarization of the mitochondrial membrane caused by the mitochondrial permeability transition (MPT) [74–76]. The molecular details of these and many other stress responses within mitochondria are not precisely understood. The compartment-specific perturbation tool used here and others that are complementary [77] may provide a means to better understand redox metabolism in this important organelle.

In a study of isolated mitochondria [40], Treberg et al. calculated H_2_O_2_ consumption rates and estimated steady state mitochondrial H_2_O_2_ concentrations of ≤ 484 ± 28 nM. In our present study of mitochondria within cells, the kinetic model says that the consumption rate of H_2_O_2_ is expected to be the same at steady-state as the generation rates of H_2_O_2_ via OxPhos and the DAAO system. With the concentration of Prx3 is taken as 62 μM within mitochondria, we calculated steady-state H_2_O_2_ concentrations of 3.4–230 nM with a generation rate from OxPhos of 4 μM/s and 0 ≤ k_DAAO_ ≤ 47 μM/s. Notably, our range in intact cells, even with extreme k_DAAO_ perturbations, is lower than the upper bound for isolated mitochondria. Our prediction of basal, steady-state concentrations of 2–4 nM H_2_O_2_ in the mitochondria of HeLa cells, with the range dependent on Prx3 concentrations from 48–110 μM, is also lower than the previously predicted value of 40 nM [20]. This previous estimate was derived using parameters for a faster respiring cell type, which would produce more H_2_O_2_ through OxPhos, perhaps leading to higher basal H_2_O_2_ concentrations. Our findings suggest the utility of measuring Prx oxidation as a marker of H_2_O_2_ concentrations. Other groups have pointed out that the Prxs could be informative biomarkers for certain cancers [78,79]. This model corroborates that idea, and demonstrates not only a relationship between Prx oxidation and H_2_O_2_ perturbation, but also Prx oxidation and the total available pool. Moving forward, this model can be used as a general framework for understanding mitochondrial H_2_O_2_ clearance, and it can be parametrized to match other cells and tissues as data become available.

## Supporting information

S1 Appendix Code, calculations, system of ODEs, further explanations. (DOCX)

S1 Fig Baseline H_2_O_2_ concentration as a function of Prx3 pool for a higher H_2_O_2_ generation rate by OxPhos (11 μM/s). A value of 4 μM/s was used to generate the figures used in the main text.
(TIF)

S2 Fig Dimer fraction as a function of Prx3 pool for a higher rate of H_2_O_2_ generation from OxPhos (11 μM/s). A value of 4 μM/s was used to generate the figures used in the main text.
(TIF)

S3 Fig Baseline concentrations of reduced and oxidized isoforms of major antioxidant species for a fixed pool of Prx3 (62 μM) and a higher rate of H_2_O_2_ generation by OxPhos (11 μM/s). A value of 4 μM/s was used to generate the figures used in the main text.
(TIF)

S4 Fig Additive intensities of all Prx3 bands (reduced and oxidized) as a function of D-ala concentration (0–24 mM) and time of increased H2O2 generation in the mitochondria (15 min.- 1 hr.). Band intensities were normalized to the endogenous protein used as a loading control (Hsp60) and each data point represents an independent replicate.
(TIF)

S5 Fig Full Western blot image from Fig 5 stained for Prx2 and Hsp60, visualized using IRDye680, showing samples from 15 min of generation, 0–25 mM D-ala (left to right). (TIF)

S6 Fig Full Western blot image from Fig 5 stained for Prx3, visualized using IRDye800, showing samples from 15 min of generation, 0–25 mM D-ala (left to right). (TIF)

S7 Fig Corresponding stain-free total protein image of 15 min Western blot. (TIF)

S8 Fig Full Western blot image from Fig 5 stained for Prx2 and Hsp60, visualized using IRDye680, showing samples from 30 min of generation, 0–25 mM D-ala (left to right).
(TIF)

S9 Fig Full Western blot image from Fig 5 stained for Prx3, visualized using IRDye800, showing samples from 30 min of generation, 0–25 mM D-ala (left to right).
(TIF)

S10 Fig Corresponding stain-free total protein image of 30 min Western blot. (TIF)

S11 Fig Full Western blot image from Fig 5 stained for Prx2 and Hsp60, visualized using IRDye680, showing samples from 1 hr of generation, 0–25 mM D-ala (left to right).
(TIF)

S12 Fig Full Western blot image from Fig 5 stained for Prx3, visualized using IRDye800, showing samples from 1 hr of generation, 0–25 mM D-ala (left to right).
(TIF)

S13 Fig Corresponding stain-free total protein image of 1 hr Western blot. (TIF)

S14 Fig Comparison of simulations from Fig 6 with experimental data from Fig 5. (TIF)
