# Supplementary material for: Kinetic modeling of H2O2 dynamics in the mitochondria of HeLa cells
Source: PLoS Comput Biol. 2020 Sep 14;16(9):e1008202. doi: 10.1371/journal.pcbi.1008202 (PMC7515204; doi:10.1371/journal.pcbi.1008202)
Supplement: S1 Appendix — (DOCX) [file pcbi.1008202.s001.docx]

**S1 Appendix**

**Matlab code**

The baseline model is accessible:

<https://github.com/sikes-lab/HeLa-mito/blob/master/runPeroxideClearanceModel_mito_Srx.m>

and copied below for explanation of numbered parameters and equations used to represent reactions.

Code for predicting basal conditions:

function [out,C] = runPeroxideClearanceModel_mito_Srx

%baseline model for hydrogen peroxide reaction network in the mitochondria

%Kassi T. Stein

%June 2018

%The overall function performs a parameter sweep over possible

%concentrations of Prx3 and solves for the baseline concentrations of 28

%different mitochondrial species, based on a constant H_2_O_2_ source from

%OxPhos. The function outputs are "C" a vector of Prx3 concentrations from

%the parameter sweep and "out" a cell array containing a matrix of the

%output from each iteration of the function in each cell.

out=cell(1,10);

C=linspace(48,110,10);

for m=1:length(out)

[OUT]=PeroxideClearanceModel_HeLa_mito(C(m),m);

out{m}=OUT;

end

end

function out = PeroxideClearanceModel_HeLa_mito(C,q)

% Model for baseline hydrogen peroxide consumption

% Kassi T. Stein

% June 2018

% model takes as input a concentration of Prx3 and an iteration number (for

% making the Excel file output)

% model outputs a matrix "out" where the first column is time and each

% susequent column is each component of the vector x (x1, x2,...,x28)

% the model also outputs and Excel file where each iteration of the

% parameter sweep is its own worksheet (make sure to specify a filename)

% Define model parameters

k = zeros(29,1);

% Intracellular peroxide production

k(1) = 4; %uM/s OCR est

% GPx1red reacting with H2O2

k(2) = 60; % uM^-1*s^-1

% GPx1ox reacting with GSH

k(3) = 4e-2; % uM^-1*s^-1

% GPx-SSG reacting with GSH

k(4) = 10; % uM^-1*s^-1 (assuming same for both isoforms)

% Km of NADP+

k(5) = 57; % uM

% Prx3-SH oxidized by H2O2

k(6) = 20; % uM^-1*s^-1

% Prx3-SOH over-oxidized by H2O2

k(7) = 1.4e-2; % uM^-1*s^-1

% Reduction of overoxidized Prx3 by Srx enzyme

k(8) = 3e-3; % uM^-1*s^-1

% Self-catalyzed disulfide formation of Prx3-SS from Prx3-SOH

k(9) = 20; % s^-1

% Prx3 is reduced by thioredoxin

k(10) = 2.2e-1; % uM^-1*s^-1

% Auto-oxidation of GSH

k(11) = 7.4e-05; % s^-1

% Pr-SH oxidized by H2O2

k(12) = 1e-4; % uM^-1*s^-1

% Pr-SOH glutathionylated by GSH

k(13) = 1.2e-1; % uM^-1*s^-1

% Grx2-SH de-glutathionylates Protein-SSG

k(14) = 1.2e-2; % uM^-1*s^-1

% GSH de-glutathionylates Grx2-SSG

k(15) = 3.7e-2; % uM^-1*s^-1

% Pr-(SH)2 oxidized by H2O2

k(16) = 1e-4; % uM^-1*s^-1

% Pr-SS reduced by Trx

k(17) = 1e-4; % uM^-1*s^-1

% GSSG reduced by GR

k(18) = 3.2; % uM^-1*s^-1

% Oxidized Thioredoxin reduced by TrxR

k(19) = 20; % uM^-1*s^-1

% Regeneration of NADPH

k(20) = 3.75e2; % uM/s

% GSH import

k(21) = 4.8e-1; % uM/s

% GSH export + degradation

k(22) = 3.2e-2; % uM/s

%Prx5-SH oxidized by H2O2

k(23) = 3e-1; %uM^-1 s^-1

%Prx5-SOH auto-catalyzes to Prx5-SS

k(24) = 14.7; %s^-1

%Prx5-SS reduced by Trx2

k(25) = 2; % uM^-1 s^-1

%GPx4red oxidized by H2O2

k(26) = 4.8e-2; % uM^-1 s^-1

%Gpx4ox reacting with GSH

k(27) = 2e-2; % uM^-1 s^-1

%DAAO producing H2O2

k(28) = 0; %uM/s (we'll use this in the perturbation case)

%Srx import

k(29) = 1.23e-5; %uM/s

%%

% Defining initial Conditions (Concentrations in uM = micromoles/Liter)

x0 = zeros(28,1);

x0(1) = .484; %H2O2 (this is a placeholder, we'll solve for it later)

x0(2) = 1.5e-2; %Gpx1red

x0(5) = 5e3; % GSH

x0(6) = 1.78; % GSSG

x0(7) = C; % Prx3-SH

x0(11) = 7.7; % Trx2-SH

x0(12) = 7.54e-2; % Trx2SS

x0(13) = 1e-3; % Pr-SH

x0(14) = x0(13)*(.5/100); % Pr-SOH

x0(15) = x0(13)*(.5/100); % Pr-SSG

x0(16) = 1; %Grx2-SH

x0(17) = x0(16)*(.5/100); % Grx-SSG

x0(18) = 1.09e3; % Pr-(SH)2

x0(20) = 30; % NADPH

x0(21) = 3.0e-1; % NADP+

x0(22) = 14; %Prx5-SH

x0(25) = 0.23; %Gpx4red

x0(28) = 8.78e-3; %Srx

% Specify baseline H2O2 concentration

initial_H2O2 = k(1)/(k(6)*x0(7));

x0(1) = initial_H2O2;

% Solve for expected initial conditions for GPXo and GPX-SG

x0(3) = x0(2)/(k(3)*x0(5)/k(2)/initial_H2O2+1+k(2)/k(4)); % GPXo

x0(4) = x0(2)/(k(4)*x0(5)/k(2)/initial_H2O2+k(4)/k(3)+1); % GPX-SG

x0(2) = x0(2) - x0(3) - x0(4); % Resolving for GPXr based on molar balance

% Solve for expected initial conditions for different oxidation states

% of Prx3

x0(8) = x0(7)/(k(9)/k(6)/initial_H2O2+1+k(9)/k(10)/x0(11)+k(7)*initial_H2O2/k(8)/x0(28));% Prx-SOH

x0(9) = x0(7)*k(7)*initial_H2O2/(k(8)*x0(28)*(k(9)/k(6)/initial_H2O2+1+k(9)/k(10)/x0(11)+k(7)*initial_H2O2/k(8)/x0(28))); % Prx-SOOH

x0(10) = x0(7)*k(9)/(k(10)*x0(11)*(k(9)/k(6)/initial_H2O2+1+k(9)/k(10)/x0(11)+k(7)*initial_H2O2/k(8)/x0(28))); % Prx-SS

x0(7) = x0(7) - x0(8) - x0(9) - x0(10); % Resolving for Prx-SH based on molar balance

% Solve for expected initial conditions for different oxidation states

% of Pr-SH

x0(14) = x0(13)/(k(13)*x0(5)/k(12)/initial_H2O2+k(13)*x0(5)/k(14)/x0(16)+1); % Pr-SOH

x0(15) = x0(13)/(k(14)*x0(16)/k(12)/initial_H2O2+1+k(14)*x0(16)/k(13)/x0(5)); % Pr-SSG

x0(13) = x0(13) - x0(14) - x0(15); % Resolving for Pr-SH based on molar balance

% Solve for expected initial condition for oxidized Pr-(SH)2, then use

% molar balance to resolve for Pr-(SH)2

x0(19) = x0(18)/(1+k(17)*x0(11)/k(16)/initial_H2O2); % Pr-SS

x0(18) = x0(18) - x0(19);

% Solve for expected initial condition for oxidized Grx-SSG, then use

% molar balance to resolve for Grx-SH

x0(17) = k(16)/(1+k(15)*x0(5)/k(14)/x0(15));

x0(16) = x0(16) - x0(17);

%solve for expected initial conditions of Prx5 states, then use molar

%balance to find Prx5-SH remaining

x0(23) = k(23)*x0(22)*initial_H2O2/k(24); %Prx5-SOH

x0(24) = k(23)*x0(22)*initial_H2O2/(k(25)*x0(11)); %Prx5-SS

x0(22) = x0(22)-x0(23)-x0(24); %Prx5-SH

% Solve for expected initial conditions for GPX4o and GPX4-SG

x0(26) = x0(26)/(k(27)*x0(5)/k(26)/initial_H2O2+1+k(26)/k(4)); % GPX4o

x0(27) = x0(26)/(k(4)*x0(5)/k(26)/initial_H2O2+k(4)/k(27)+1); % GPX4-SG

x0(25) = x0(25) - x0(26) - x0(27); % Resolving for GPX4r based on molar balance

%%

% Solver Parameters

ti = 0; % start time

tf = 5; % stop time (s)

%ode15s is a stiff ODE solver, so will use variable time steps at short vs.

%long times; only need to specify start and end time, not time steps

tspan = [ti tf];

%force the function to be always positive

negspan = [1:28];

options=odeset('AbsTol',1E-10,'RelTol',1E-4,'NonNegative',negspan);

% Integration

tic

[t,x]=ode15s(@crank,tspan,x0,options,k);

toc

out = [t, x];

filename = 'HeLa_srxn1_notrx.xlsx';

xlswrite(filename,out,q,'B3');

%%

% Description of derivatives

function dxdt = crank(t, x, k);

dxdt= x; % setting up vector containing derivatives

dxdt(1) = k(1) - k(2)*x(2)*x(1) - k(6)*x(7)*x(1) - k(7)*x(8)*x(1)...

- k(12)*x(13)*x(1) - k(16)*x(18)*x(1)-k(23)*x(22)*x(1)-k(26)*x(25)*x(1); % H2O2

dxdt(2) = -k(2)*x(2)*x(1) + k(4)*x(4)*x(5); % GPX1red

dxdt(3) = k(2)*x(2)*x(1) - k(3)*x(3)*x(5); % GPX1ox

dxdt(4) = k(3)*x(3)*x(5) - k(4)*x(4)*x(5); % GPX1-SG

dxdt(5) = -k(3)*x(3)*x(5) - k(4)*x(4)*x(5) - 2*k(11)*x(5)...

- k(13)*x(14)*x(5) - k(15)*x(17)*x(5) + 2*k(18)*x(6)*x(20) + k(21) - k(22)...

- k(27)*x(26)*x(5) - k(4)*x(27)*x(5); % GSH

dxdt(6) = k(4)*x(4)*x(5) + k(11)*x(5) + k(15)*x(17)*x(5) + k(4)*x(27)*x(5) - k(18)*x(6)*x(20); % GSSG

dxdt(7) = -k(6)*x(7)*x(1) + k(10)*x(10)*x(11); % Prx3-SH

dxdt(8) = k(6)*x(7)*x(1) - k(7)*x(8)*x(1) + k(8)*x(9)*x(28) - k(9)*x(8); % Prx-SOH

dxdt(9) = k(7)*x(8)*x(1) - k(8)*x(9)*x(28); % Prx-SOOH

dxdt(10) = k(9)*x(8) - k(10)*x(10)*x(11); % Prx3-SS

dxdt(11) = -k(10)*x(10)*x(11) - k(17)*x(19)*x(11) - k(25)*x(24)*x(11)...

+ k(19)*x(12)*x(20); % Trx-SH

dxdt(12) = k(10)*x(10)*x(11) + k(17)*x(19)*x(11) + k(25)*x(24)*x(11)...

- k(19)*x(12)*x(20); % Trx-SS

dxdt(13) = -k(12)*x(13)*x(1) + k(14)*x(16)*x(15); % Pr-SH

dxdt(14) = k(12)*x(13)*x(1) - k(13)*x(14)*x(5); % Pr-SOH

dxdt(15) = k(13)*x(14)*x(5) - k(14)*x(16)*x(15); % Pr-SSG

dxdt(16) = k(15)*x(17)*x(5) - k(14)*x(16)*x(15); % Grx-SH

dxdt(17) = k(14)*x(16)*x(15) - k(15)*x(17)*x(5); % Grx-SSG

dxdt(18) = -k(16)*x(18)*x(1) + k(17)*x(19)*x(11); % Pr-(SH)2

dxdt(19) = k(16)*x(18)*x(1) - k(17)*x(19)*x(11); % Pr-SS

dxdt(20) = -k(18)*x(6)*x(20) - k(19)*x(12)*x(20) + k(20)*x(21)/(k(5) + x(21)); % NADPH

dxdt(21) = k(18)*x(6)*x(20) + k(19)*x(12)*x(20) - k(20)*x(21)/(k(5) + x(21)); % NADP+

dxdt(22) = -k(23)*x(22)*x(1) + k(25)*x(24)*x(11); %Prx5-SH

dxdt(23) = k(23)*x(22)*x(1) - k(24)*x(23); %Prx5-SOH

dxdt(24) = k(24)*x(23) - k(25)*x(24)*x(11); %Prx5-SS

dxdt(25) = -k(26)*x(25)*x(1) + k(4)*x(27)*x(5); % GPX4red

dxdt(26) = k(26)*x(25)*x(1) - k(27)*x(26)*x(5); % GPX4ox

dxdt(27) = k(27)*x(26)*x(5) - k(4)*x(27)*x(5); % GPX4-SG

dxdt(28) = k(29); %Srx

end

end

The code used to predict increases in the H_2_O_2_ generation rate due to the action of mitochondrially localized DAAO is similarly accessible:

<https://github.com/sikes-lab/HeLa-mito/blob/master/runPeroxideClearanceModel_mito_DAAOwSrx.m>

## Calculations to obtain parameter values

The rate of H_2_O_2_ generation from the electron transport chain (ETC) due to oxidative phosphorylation (OxPhos) was calculated based on oxygen consumption rate (OCR) data and H_2_O_2_ production rate data from respiring mitochondria isolated from rat liver [1,2]. We reconciled the data from these two sources in order to estimate this important parameter.

The reported OCR from HeLa cells was $200\frac{\text{amol}}{\text{ng-protein*s}}$ [1]. HeLa cell protein concentration was reported as $2x10^{5}$ mg/L [3], so we used this number to convert the reported OCR to 40 µM/s. Up to 10% of the O_2_ consumption can be assumed to get converted to H_2_O_2_ [4], giving a value of 4 µM/s H_2_O_2_ produced by OxPhos.

$$200\frac{\text{amol}}{\text{ng-protein*s}}*2x10^{11}\frac{\text{ng-protein}}{\text{L}}*10\%=4x10^{13}\frac{\text{aM}}{\text{s}}=4\frac{\text{μM}}{\text{s}}$$

The data reported by Treberg *et al.* provided a range of possible H_2_O_2_ production rates, as measured extramitochondrially. Based on the methodology presented in their paper, any measured extramitochondrial H_2_O_2_ rates are lower than what is actually produced because some H_2_O_2_ has already been consumed by the mitochondrial antioxidant network [2]. They propose the following equation:

$$\frac{V_{p}}{k}=\left[H_{2}O_{2}\right]_{ss}$$

where $V_{p}$ is the rate of H_2_O_2_ production, *k* is the first order rate constant that describes the mitochondrial reaction network, and $\left[H_{2}O_{2}\right]_{ss}$ is the steady state concentration of H_2_O_2_. For our system, *k* is the pseudo-first order rate constant of Prx3, equal to 1200 s^-1^, as this is the largest kinetic constant in our network. Applying our rate constants with the reported $\left[H_{2}O_{2}\right]_{ss}$ of $1\frac{\text{nmol}}{\text{min-mg}}$ [2] we can calculate an H_2_O_2_ production rate of 5 µM/s. This is the same order of magnitude as the 4 µM/s calculated by using the OCR approach above, and we took the number associated with the OCR data as this was measured from HeLa cells rather than rat liver mitochondria.

To convert protein copy number reported by Itzhak *et al.* [3] to a mitochondrial protein concentration, the following calculations were carried out:

$$c_{i}=\frac{n}{N_{A}N_{mito}V_{mito}}$$

where $c_{i}$ is the concentration of protein *i*, *n* is the protein copy number associated with all the mitochondria in a single cell (given in the proteomics dataset), $N_{A}$ is Avogadro’s number, $N_{mito}$ is the number of mitochondria in a single cell (383 – 882) [5], and $V_{mito}$ is the volume of one mitochondrion (0.29 µm^3^) [6]. If the protein was not reported in the organelle-specific dataset, but is known to localize in the mitochondria (the glutathione peroxidases) then its copy number was pulled from the proteome-wide dataset and then the mitochondrial concentration was still calculated in this manner.

The initial concentration of H_2_O_2_ used in the model at *t = 0* was calculated based on the consumption by Prx3:

$$c_{H_{2}O_{2},i}=\frac{k_{1}}{k_{6}c_{Prx3-SH,T}}$$

The magnitude of the Prx3-SH term is much larger than any other terms we might have included in this calculation, so this reduced term is sufficient to initialize the model.

For related isoforms of proteins, the pseudo-steady state assumption was used to find the initial concentrations of each species [7]. For the Prx3 isoforms, those calculations were as follows:

$$c_{\Pr x3-SOH,i}=\frac{c_{\Pr x3-SH,T}}{\frac{k_{9}}{k_{6}c_{H_{2}O_{2},i}}+1+\frac{k_{9}}{k_{10}c_{Trx2-SH,i}}+\frac{k_{7}c_{H_{2}O_{2},i}}{k_{8}c_{Srx,i}}}$$

$$c_{\Pr x3-SOOH,i}=\frac{k_{7}c_{\Pr x3-SH,T}c_{H_{2}O_{2},i}}{k_{8}c_{Srx,i}\left(\frac{k_{9}}{k_{6}c_{H_{2}O_{2},i}}+1+\frac{k_{9}}{k_{10}c_{Trx2-SH,i}}+\frac{k_{7}c_{H_{2}O_{2},i}}{k_{8}c_{Srx,i}}\right)}$$

$$c_{\Pr x3-SS,i}=\frac{k_{9}c_{\Pr x3-SH,T}}{k_{10}c_{Trx2-SH,i}\left(\frac{k_{9}}{k_{6}c_{H_{2}O_{2},i}}+1+\frac{k_{9}}{k_{10}c_{Trx2-SH,i}}+\frac{k_{7}c_{H_{2}O_{2},i}}{k_{8}c_{Srx,i}}\right)}$$

$$c_{\Pr x3-SH,i}=c_{\Pr x3-SH,T}-c_{\Pr x3-SOH}-c_{\Pr x3-SOOH}-c_{\Pr x3-SS}$$

The same strategy was followed for the other protein families for consistent initialization.

## System of ODEs

$$\frac{dc_{Srx}}{dt}=k_{29}$$

**Quantifying effects of uncertainty in parameters on model predictions**

(codes available in https://github.com/sikes-lab/HeLa-mito/)

**Sensitivity analysis**

Sensitivity analysis, as described in the main text, was conducted using the following equations:

$s_{i}\left(t\right)=\frac{\partial C_{j}\left(t\right)}{\partial k_{i}}=\frac{C_{j}\left(k_{i}+\Delta k_{i},t\right)-C_{j}\left(k_{i},t\right)}{\Delta k_{i}}$ (S37)

where $s_{i}$ is the sensitivity corresponding to parameter $k_{i}$ and $C_{j}$ is the concentration of the species of interest. Parameters were perturbed by 10% to reflect an estimate of typical experimental error, and sensitivities were normalized to adjust for differences in orders of magnitude:

$\overline{s_{i}}\left(t\right)=\frac{\partial C_{j}\left(t\right)/C_{j}\left(t\right)}{\partial k_{i}/k_{i}}$ (S38)

These calculations, presented in Figure 3, were carried out using a code that performed the following steps:

1. Use initial conditions, x0, determined from <https://github.com/sikes-lab/HeLa-mito/blob/master/runPeroxideClearanceModel_mito_Srx.m> as inputs.

2. Vary *k* values

3. With the new *k* values, update the initial conditions as initial conditions are function of *k* values. [Lines 137 – 177]

4. Run ODE

5. Calculate sensitivity at t = 5s.

**Supplementary discussion of model structure**

One challenge in modeling the H_2_O_2_ clearance network in a mitochondrion is that detailed, quantitative information about how NADPH is regenerated in this compartment is not currently available. Several mechanisms have been proposed and demonstrated, but the quantitative contributions of each have not been established. In the absence of this compartment-specific information, we have used cytosolic NADPH regeneration rates via the pentose phosphate pathway as per S.28-S.29 above as an estimate. NADPH can be transferred indirectly via metabolite shuttles between the mitochondria and the cytosol.[8,9] Further, Fan et al., also demonstrated that NADPH generation in mitochondria from two carbon subunits could be as high as that from the pentose phosphate pathway.[10] We used sensitivity analyses to investigate the implications of this estimate. Parameters related to NADPH were not among those that had the largest impact on our predictions.

We note that we have not assumed an oligomerized state for Prx3. Within the assumptions of mass action kinetics, the concentration of the reactive cysteine is the important parameter and the system is assumed to be well-mixed.

Certain reactions that have been described in the literature are not included in this model. For example, it has been reported that peroxynitrite can oxidize Prx3.[11] However, for the system modeled here with generation rates of peroxynitrite that are orders of magnitude lower than that of H_2_O_2_, (nM/s vs µM/s) [12–14] we believe it is mathematically justified that the reaction of Prx3 with peroxynitrite can be neglected given our focus on steady state H_2_O_2_ concentrations.

Similarly, Grx2 has been reported as capable of reducing oxidized Prx3.[15] In our study, the concentration of Grx2 is much lower than that of Trx2, and Trx2 is mostly reduced under basal conditions. For our questions of interest, we deemed it reasonable to consider Trx2 as the dominant reduction pathway. Sensitivity analysis may be used to quantify the expected impact of increasing the concentration of reductants of oxidized Prx3.

For those who may use this model as a starting point to investigate other systems or questions, it is important to consider whether additional reactions should be added to the model.

Alternative treatment of parameter values may also be needed depending on the research question. For example, circadian oscillations in Prx3 hyperoxidation in mitochondria from several healthy tissues have been reported and mathematically modeled.[16,17] In our study, cultures were not synchronized and we did not model the variable H_2_O_2_ source terms and/or Srx concentrations that are hypothesized to result in this oscillatory behavior. With respect to the cancer killing hypothesis [18,19], H_2_O_2_ steady states in the mitochondria of tumors would need to be higher or more readily perturbed above a toxic threshold than the oscillations in the healthy tissues mentioned for redox therapies to function safely and effectively.

**Monte Carlo parameter sampling**

As described in the text, Monte Carlo parameter sampling was used to explore ranges of important, yet uncertain, parameters. Namely, these parameters were the total concentration of Prx3 in a mitochondrion, all considered to be in the reduced Prx3-SH form at the start of a simulation, $\boldsymbol{k}_{\boldsymbol{DAAO}}$, the rate constant for generation of H_2_O_2_ by mitochondrially-targeted D-amino acid oxidase, and $\boldsymbol{k}_{\boldsymbol{efflux}}$*,* the rate constant for release of H_2_O_2_ from the mitochondria to the cytosol.

10,000 random samples were generated using a uniform distribution for each of these parameters. Sets of these 10,000 random parameter samples for a) Prx3-SH with minimum at 48 µM and maximum at 110 µM, b) $\boldsymbol{k}_{\boldsymbol{DAAO}}$ with min at 50 µM/s and max at 100 µM/s, c) $\boldsymbol{k}_{\boldsymbol{efflux}}$ with min at 50 µM/s and max at 100 µM/s, d) $\boldsymbol{k}_{\boldsymbol{DAAO}}$ with min at 50 µM/s and max at 100 µM/s, and e) $\mathbf{k}_{\mathbf{efflux}}$ with min at 0 and max at 50 µM/s are described below. These samples were used in the simulations presented in Figures 2B and 6B-F.

**References:**

1. Wagner BA, Venkataraman S, Buettner GR. The rate of oxygen utilization by cells. Free Radic Biol Med. 2011;51: 700–712. doi:10.1016/j.freeradbiomed.2011.05.024

2. Treberg JR, Munro D, Banh S, Zacharias P, Sotiri E. Differentiating between apparent and actual rates of H_2_O_2_ metabolism by isolated rat muscle mitochondria to test a simple model of mitochondria as regulators of H_2_O_2_ concentration. Redox Biol. 2015;5: 216–224. doi:10.1016/j.redox.2015.05.001

3. Itzhak DN, Tyanova S, Cox J, Borner, GHH. Global , quantitative and dynamic mapping of protein subcellular localization. eLife 2016;5: e16950. doi:10.7554/eLife.16950

4. Jones DP. Radical-free biology of oxidative stress. Am J Physiol Cell Physiol. 2008;295: C849–C868. doi:10.1152/ajpcell.00283.2008

5. Posakony JW, England JM, Attardi G. Mitochondrial growth and division during the cell cycle in HeLa cells. J Cell Biol. 1977;74: 468–491. doi:10.1083/jcb.74.2.468

6. Milo R, Phillips, R. Cell biology by the numbers. Garland Science, Taylor and Francis. 2016.

7. Fogler SH. The elements of chemical reaction engineering, 5^th^ Edition. Prentice Hall, 2016.

8. MacDonald MJ. Feasibility of a mitochondrial pyruvate malate shuttle in pancreatic islets. J. Biol. Chem. 1995; 270: 20051-20058.

9. Lewis CA, Parker SJ, Fiske BP, Mccloskey D, Gui DY, Green CR, et al. Tracing compartmentalized NADPH metabolism in the cytosol and mitochondria of mammalian cells. Mol Cell. 2014;55: 253–263. doi:10.1016/j.molcel.2014.05.008

10. Fan J, Ye J, Kamphorst JJ, Shlomi T, Thompson CB, Rabinowitz JD. Quantitative flux analysis reveals folate-dependent NADPH production. Nature. 2014;510: 298–302. doi:10.1038/nature13236

11. Inés M, Armas D, Esteves R, Viera N, Reyes AM. Rapid peroxynitrite reduction by human peroxiredoxin 3 : Implications for the fate of oxidants in mitochondria. Free Radic Biol and Med. 2018;130: 369–378. doi:10.1016/j.freeradbiomed.2018.10.451

12. Murphy MP. How mitochondria produce reactive oxygen species. Biochem J. 2009; 417: 1-13. doi:10.1042/BJ20081386

13. Valdez LB, Bombicino SS, Iglesias DE, Mikusic IR, Boveris A. Mitochondrial peroxynitrite generation is mainly driven by superoxide steady-state concentration rather than by nitric oxide steady-state concentration. Int J Mol Biol Open Access. 2018;3:56–61. doi:10.15406/ijmboa.2018.03.00051

14. Radi R. Oxygen radicals, nitric oxide and peroxynitrite: Redox pathways in molecular medicine. Proc Natl Acad Sci. USA 2018;115: 5839–5848. doi:10.1073/pnas.1804932115

15. Hanschmann E, Lonn ME, Schutte LD, Funke M, Godoy JR, Eitner S, et al. Both thioredoxin 2 and glutaredoxin 2 contribute to the reduction of the mitochondrial 2-cys peroxiredoxin Prx3. J Biol Chem. 2010;285: 40699–40705. doi:10.1074/jbc.M110.185827

16. Kil IS, Ryu KW, Lee SK, Kim JY, Chu SY, Kim JH, et al. Circadian oscillation of sulfiredoxin in the mitochondria. Mol Cell. 2015;59: 651–663. doi:10.1016/j.molcel.2015.06.031

17. Olmo M, Kramer A, Herzel H. A robust model for circadian redox oscillations. Int J Mol Sci. 2019;20: E2368. doi: 10.3390/ijms20092368

18. Trachootham D, Alexandre J, Huang P. Targeting cancer cells by ROS-mediated mechanisms: a radical therapeutic approach? Nat Rev Drug Discov. 2009;7: 579-591. doi:10.1038/nrd2803

19. Gorrini C, Harris IS, Mak TW. Modulation of oxidative stress as an anticancer strategy. Nat Rev Drug Discov. 2013;12: 931-947. doi:10.1038/nrd4002
