# Supplementary figures and images for: Kinetic modeling of H2O2 dynamics in the mitochondria of HeLa cells
Source: PLoS Comput Biol. 2020 Sep 14;16(9):e1008202. doi: 10.1371/journal.pcbi.1008202 (PMC7515204; doi:10.1371/journal.pcbi.1008202)

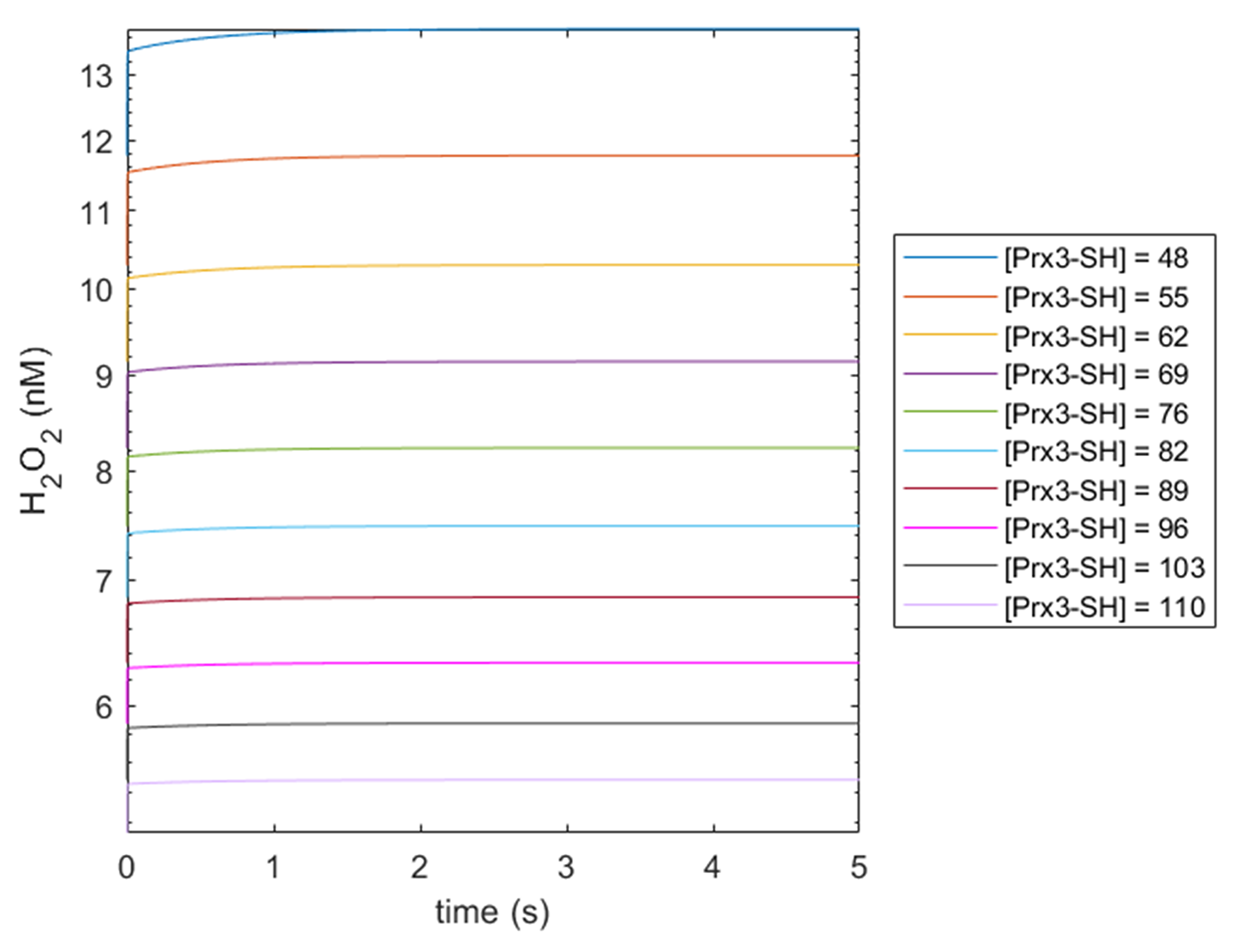

Supplement: S1 Fig — A value of 4 μM/s was used to generate the figures used in the main text. (TIF) [file pcbi.1008202.s002.tif]

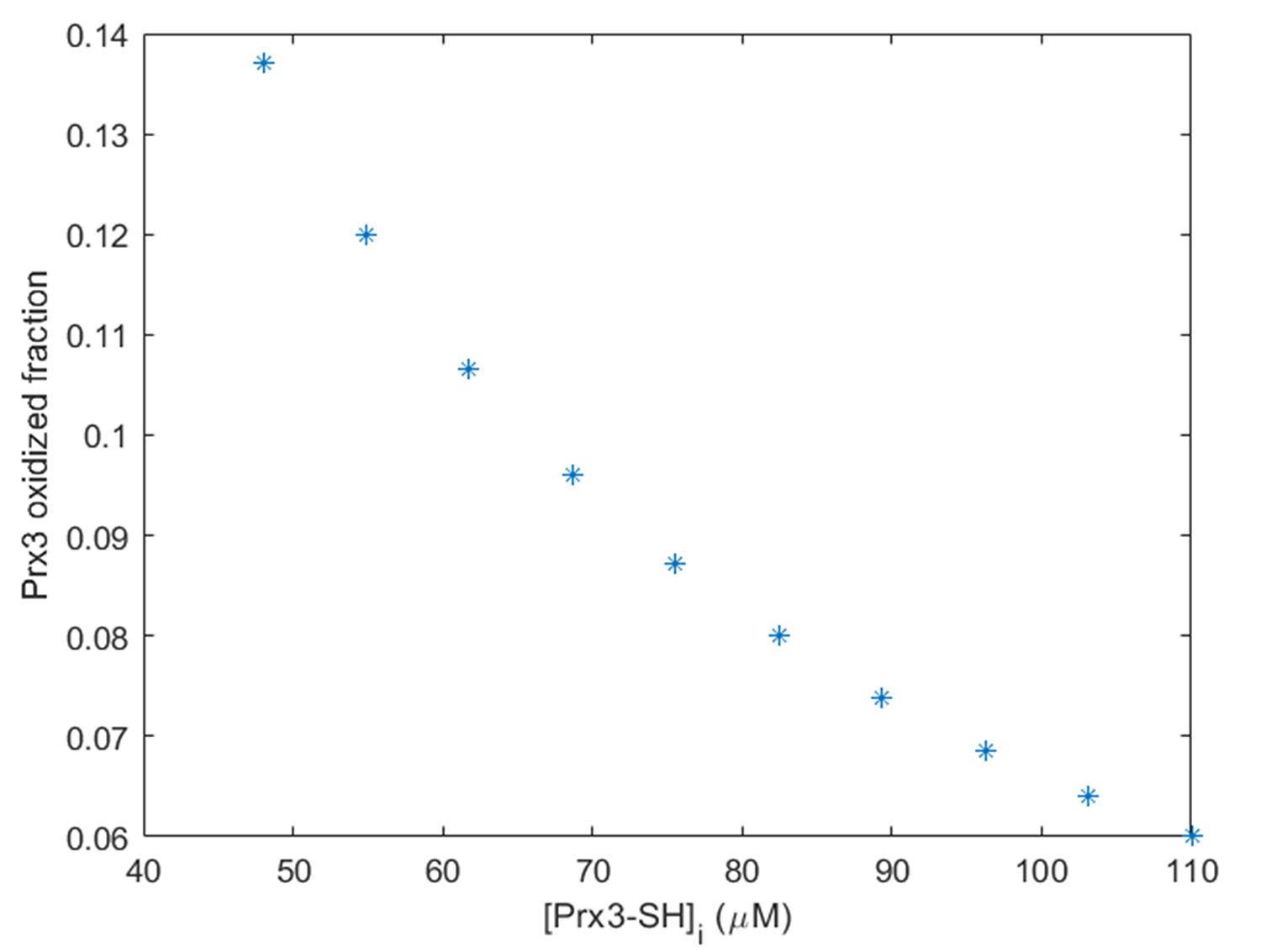

Supplement: S2 Fig — A value of 4 μM/s was used to generate the figures used in the main text. (TIF) [file pcbi.1008202.s003.tif]

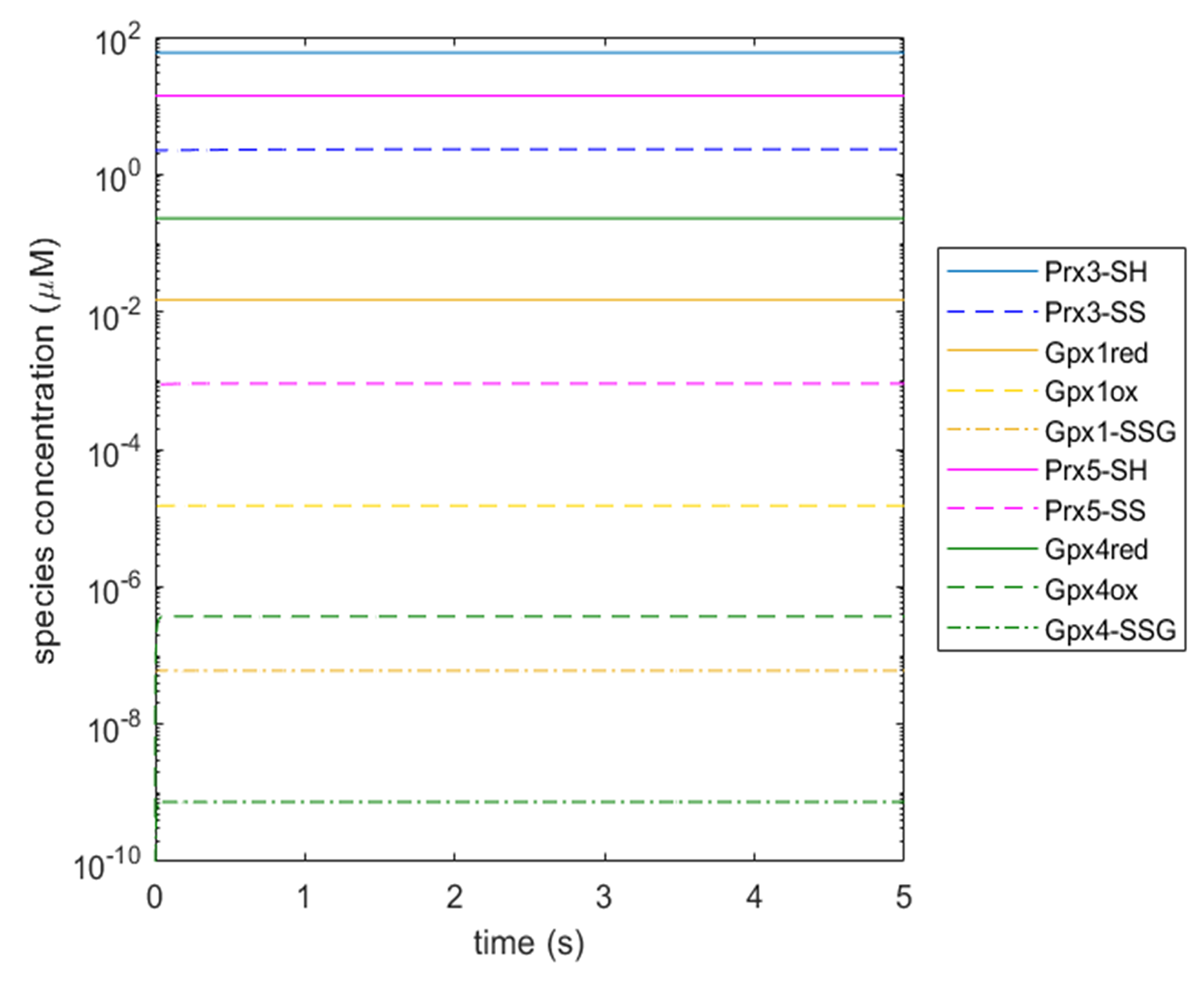

Supplement: S3 Fig — A value of 4 μM/s was used to generate the figures used in the main text. (TIF) [file pcbi.1008202.s004.tif]

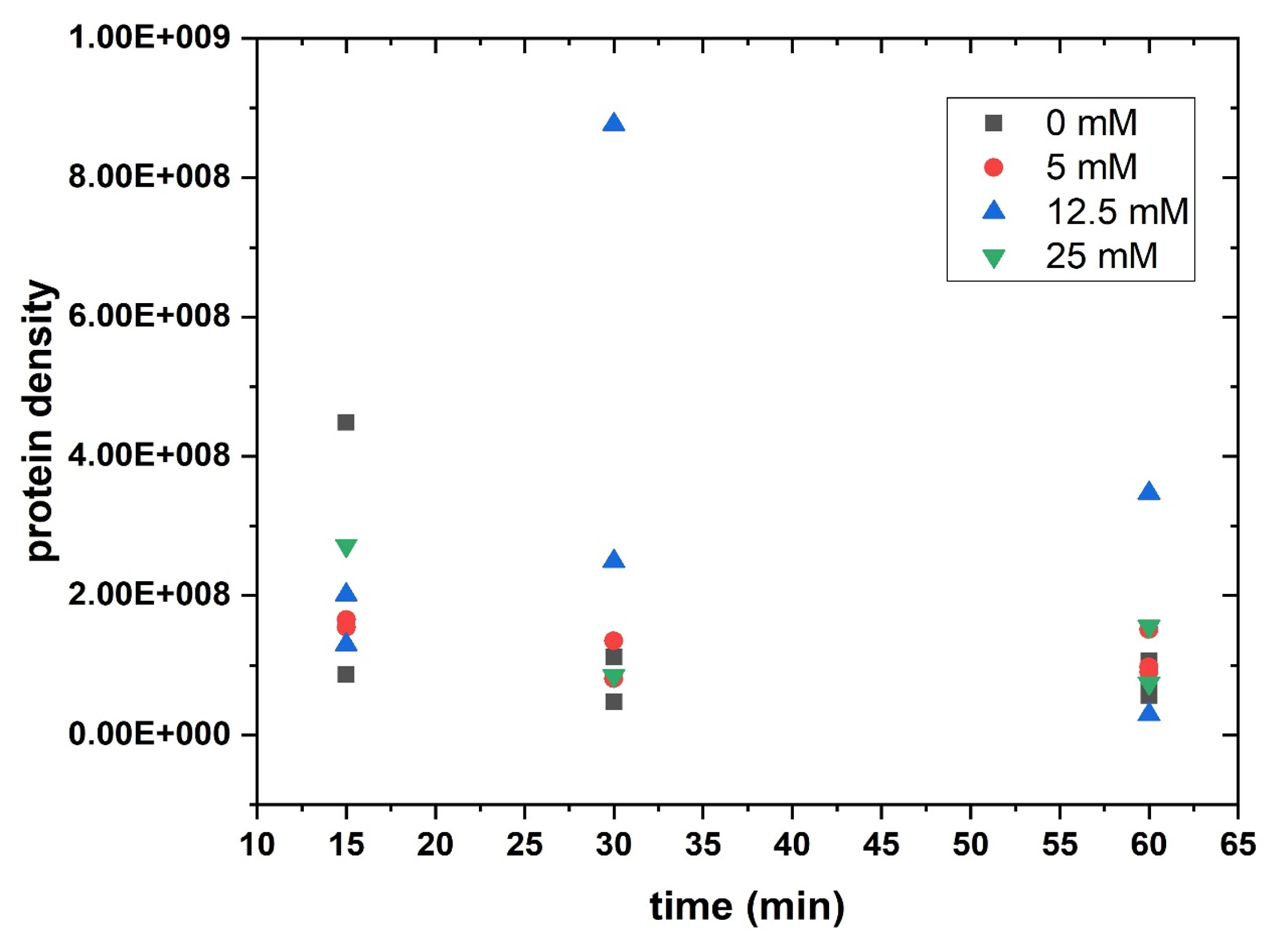

Supplement: S4 Fig — Band intensities were normalized to the endogenous protein used as a loading control (Hsp60) and each data point represents an independent replicate. (TIF) [file pcbi.1008202.s005.tif]

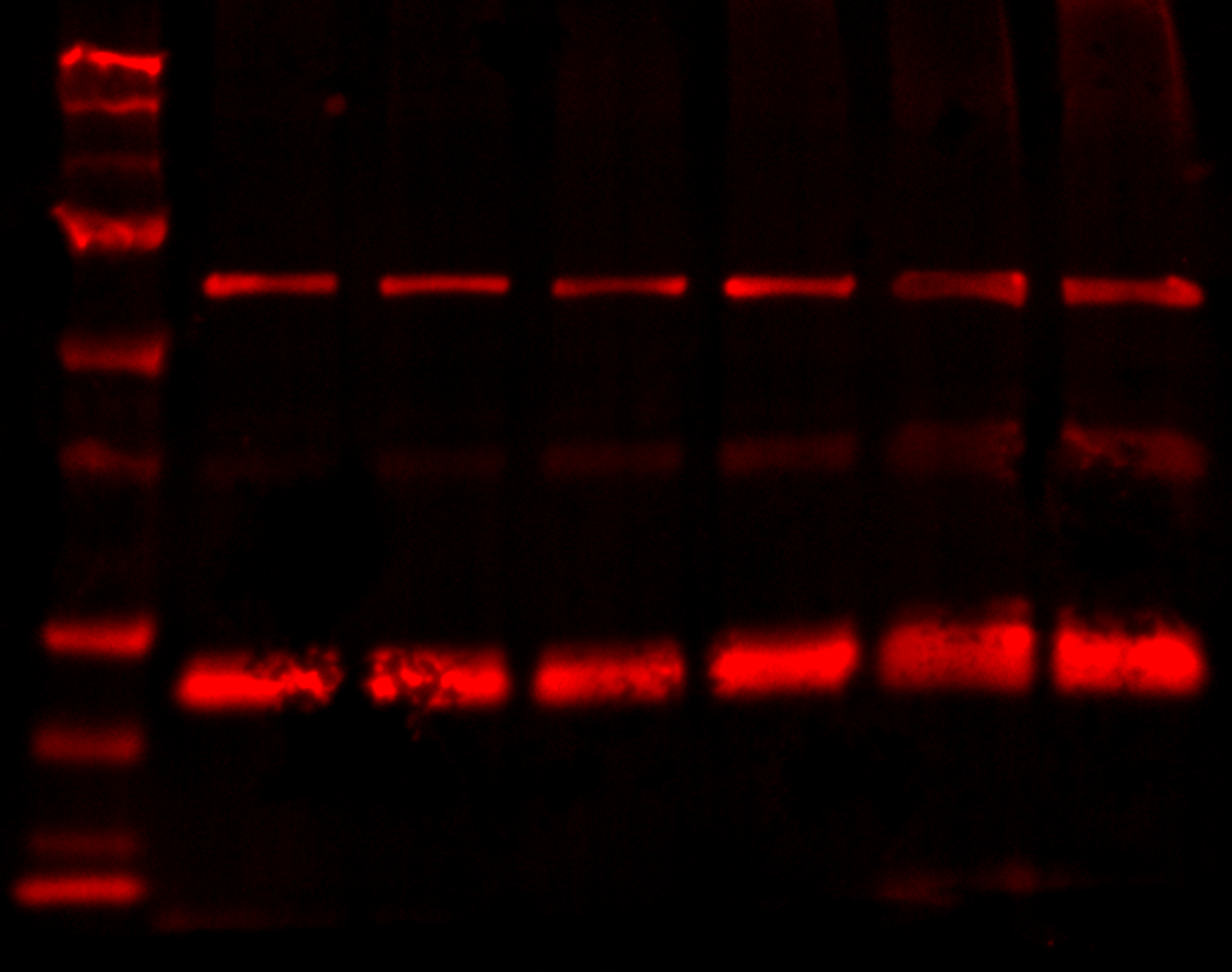

Supplement: S5 Fig — (TIF) [file pcbi.1008202.s006.tif]

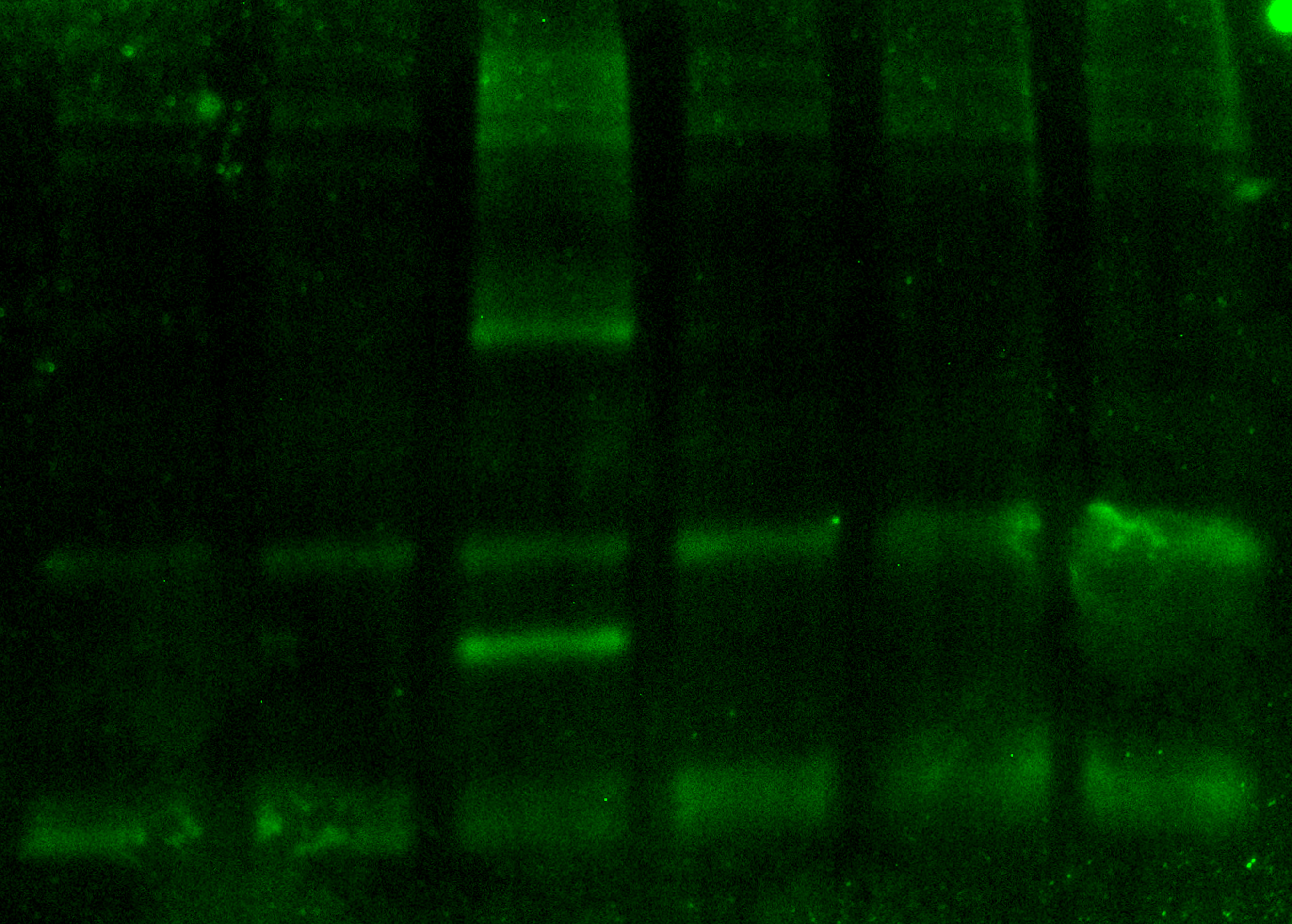

Supplement: S6 Fig — (TIF) [file pcbi.1008202.s007.tif]

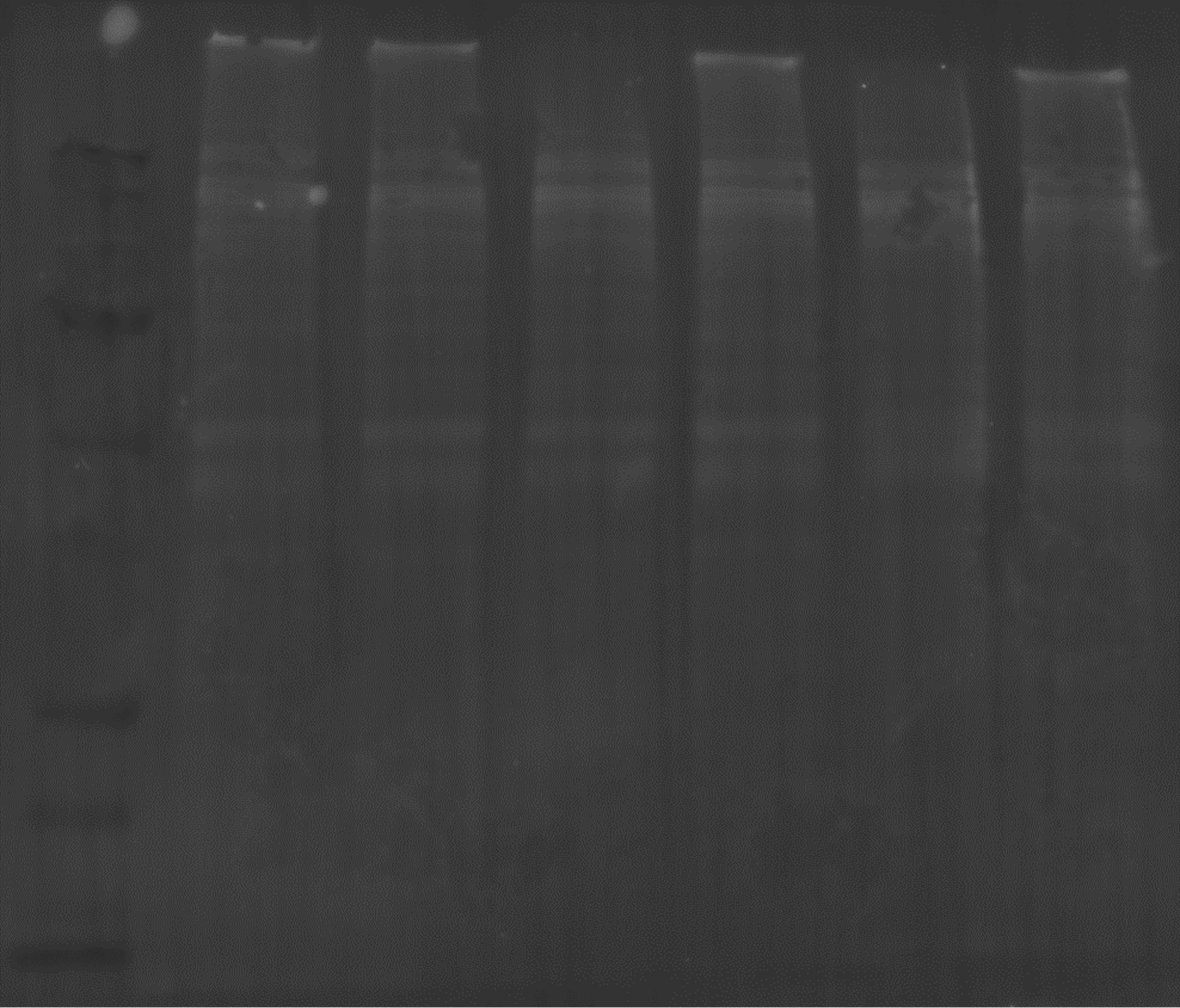

Supplement: S7 Fig — (TIF) [file pcbi.1008202.s008.tif]

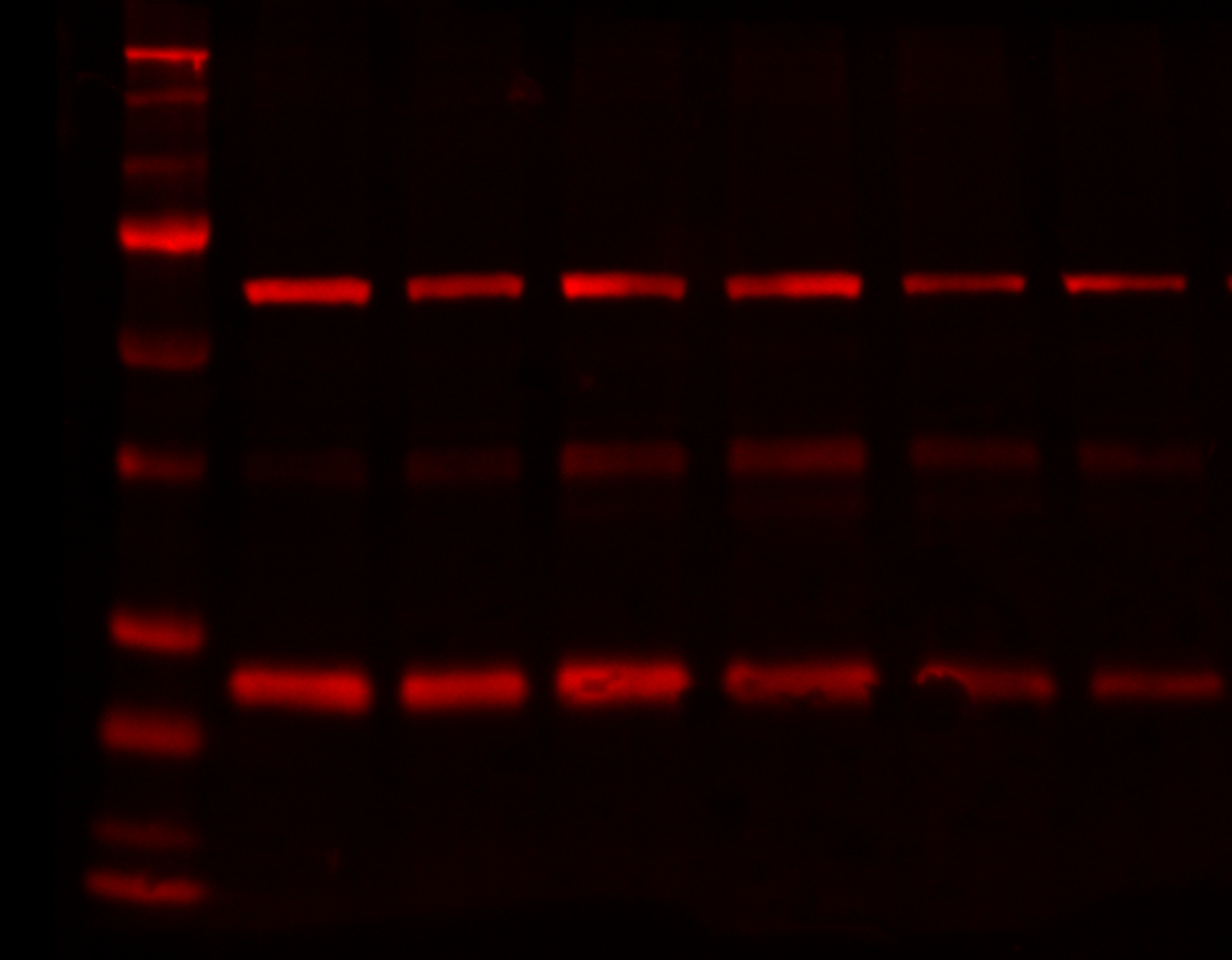

Supplement: S8 Fig — Full Western blot image from Fig 5 stained for Prx2 and Hsp60, visualized using IRDye680, showing samples from 30 min of generation, 0–25 mM D-ala (left to right). (TIF) [file pcbi.1008202.s009.tif]

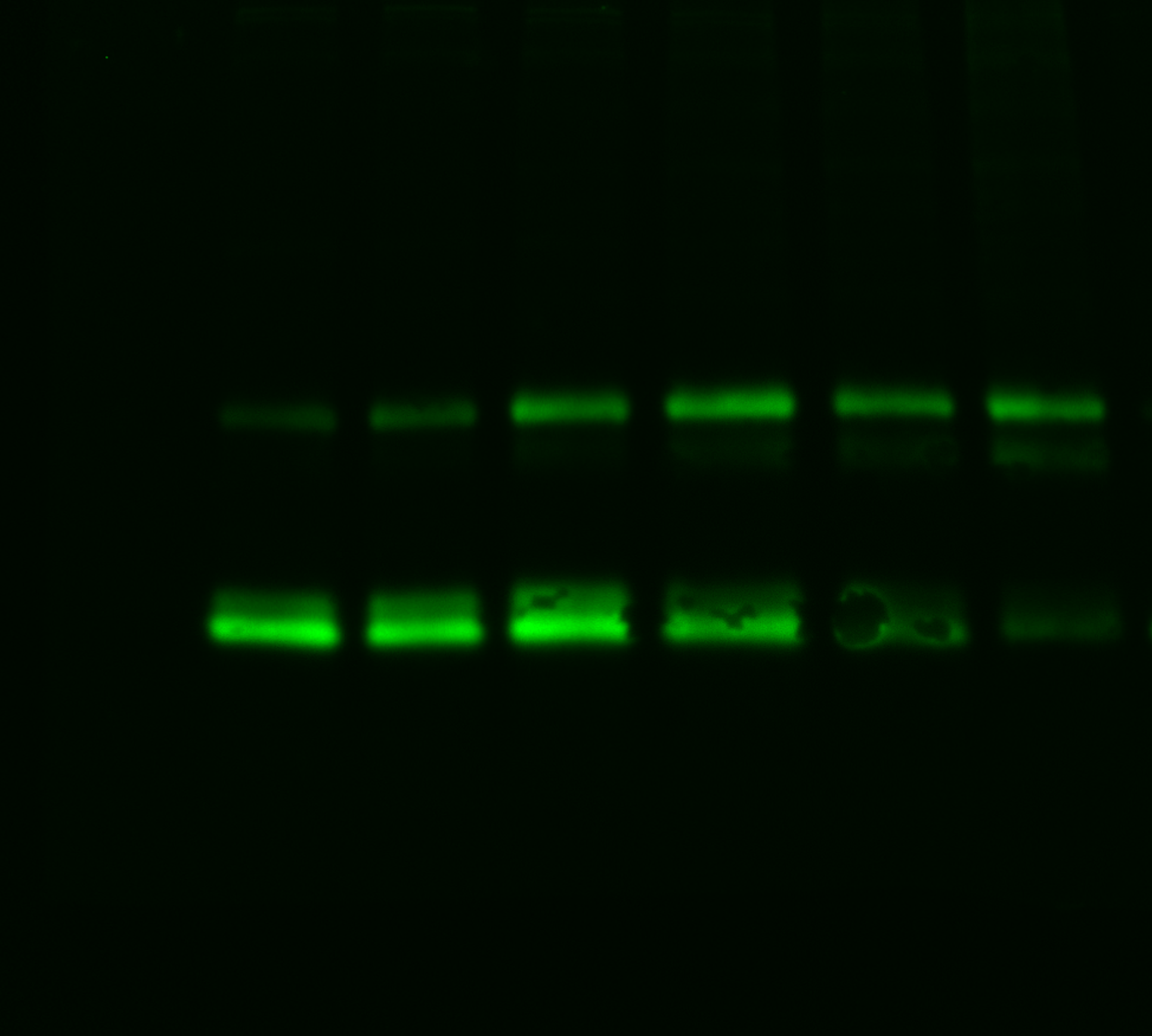

Supplement: S9 Fig — Full Western blot image from Fig 5 stained for Prx3, visualized using IRDye800, showing samples from 30 min of generation, 0–25 mM D-ala (left to right). (TIF) [file pcbi.1008202.s010.tif]

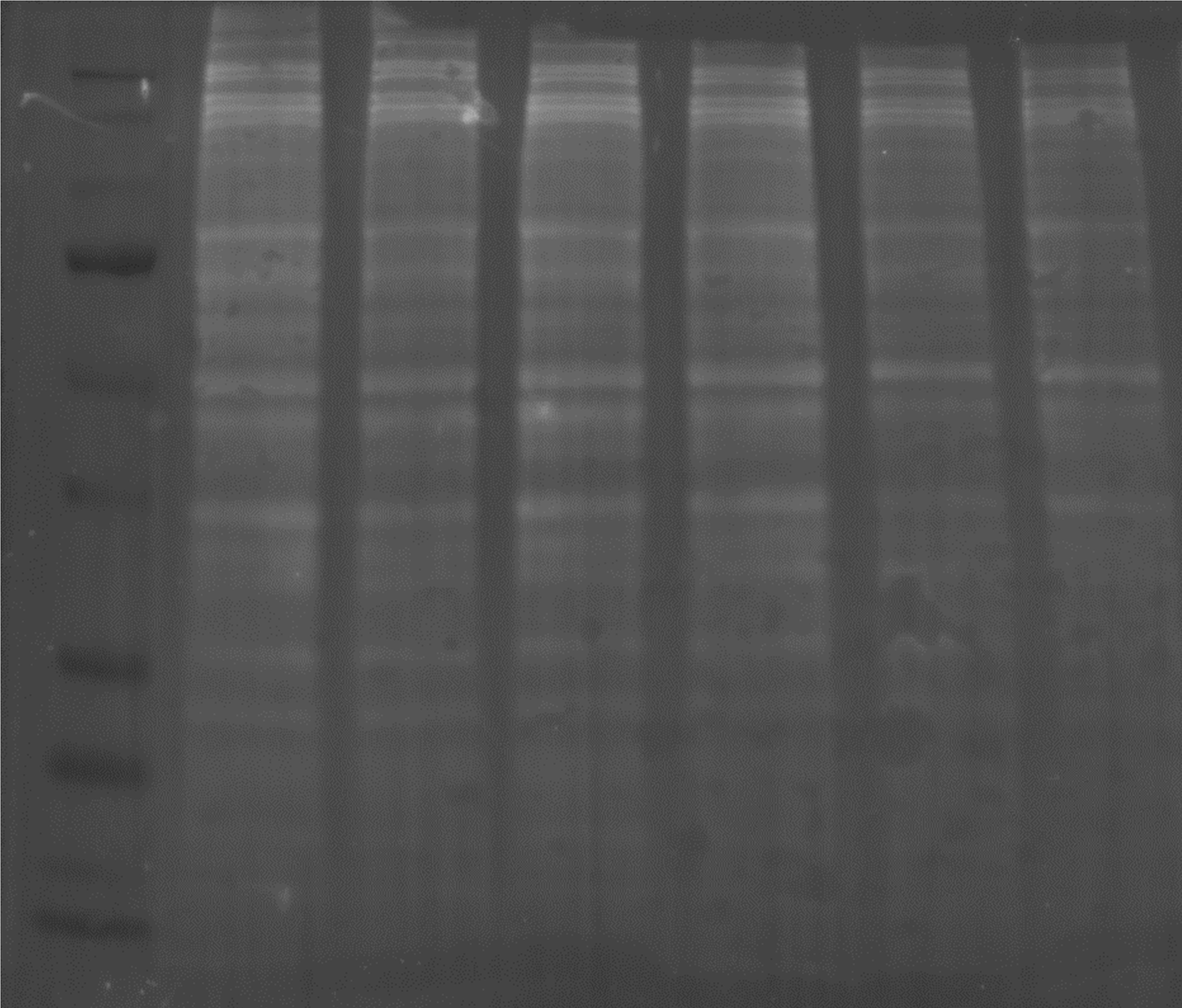

Supplement: S10 Fig — (TIF) [file pcbi.1008202.s011.tif]

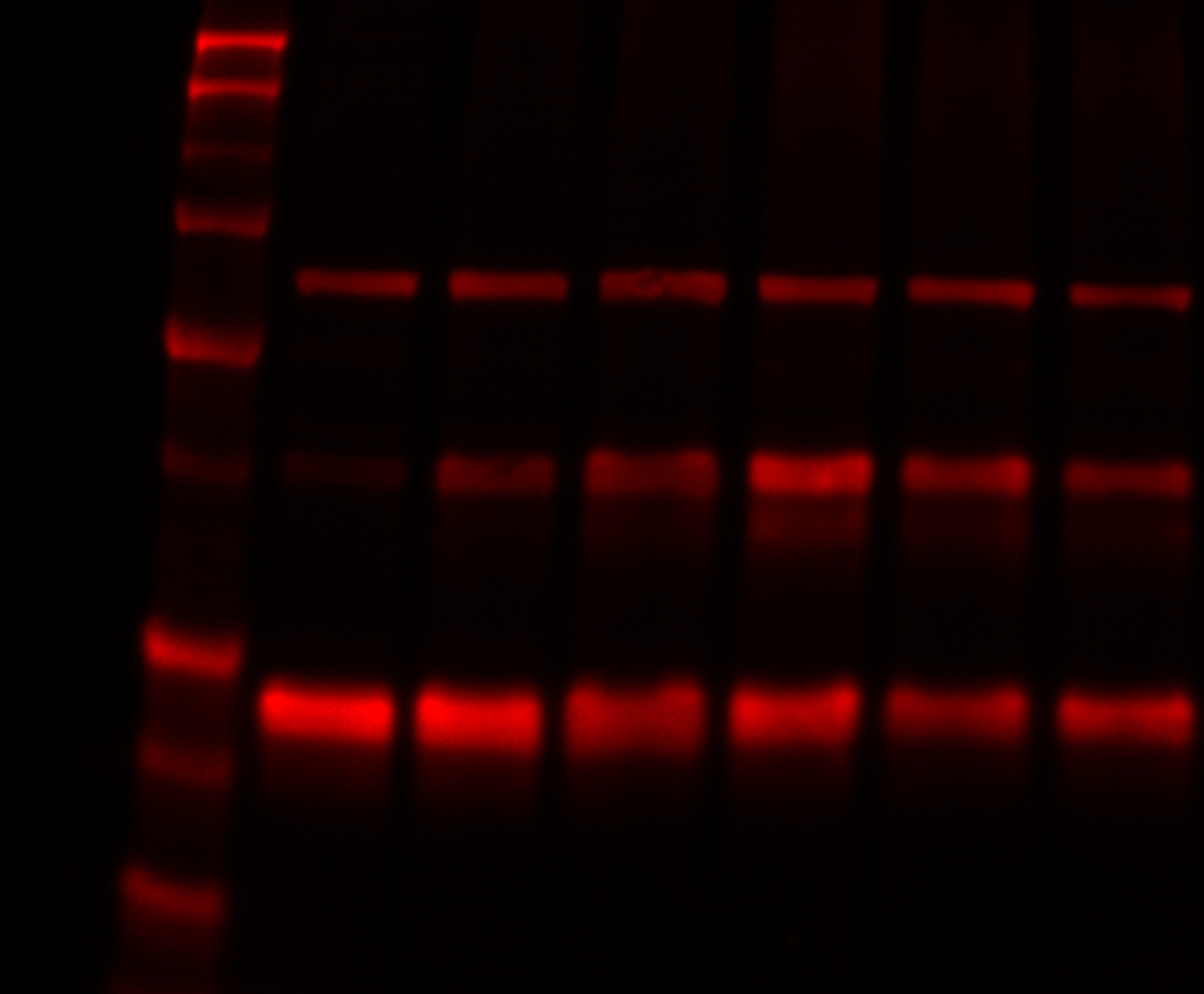

Supplement: S11 Fig — Full Western blot image from Fig 5 stained for Prx2 and Hsp60, visualized using IRDye680, showing samples from 1 hr of generation, 0–25 mM D-ala (left to right). (TIF) [file pcbi.1008202.s012.tif]

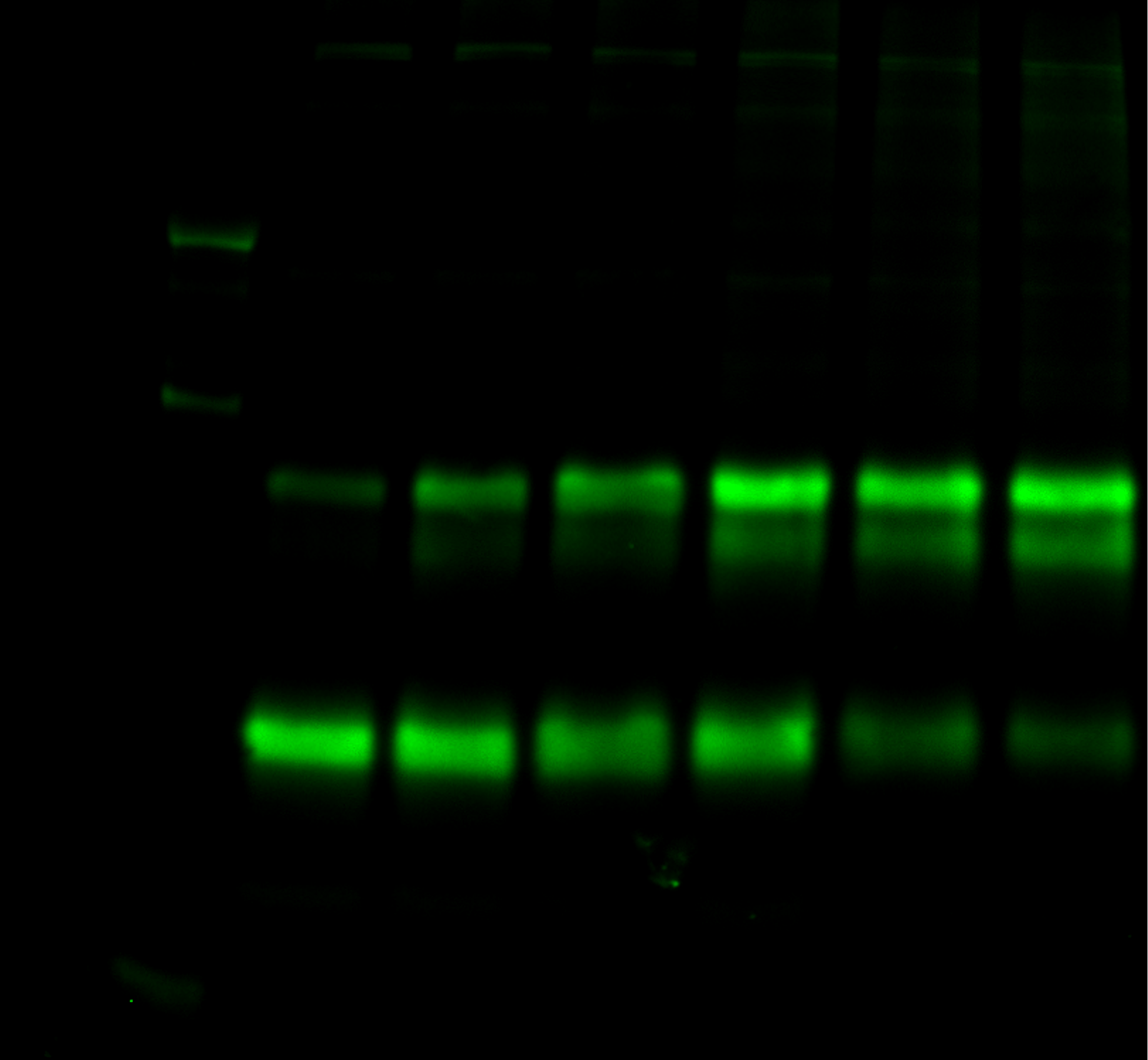

Supplement: S12 Fig — Full Western blot image from Fig 5 stained for Prx3, visualized using IRDye800, showing samples from 1 hr of generation, 0–25 mM D-ala (left to right). (TIF) [file pcbi.1008202.s013.tif]

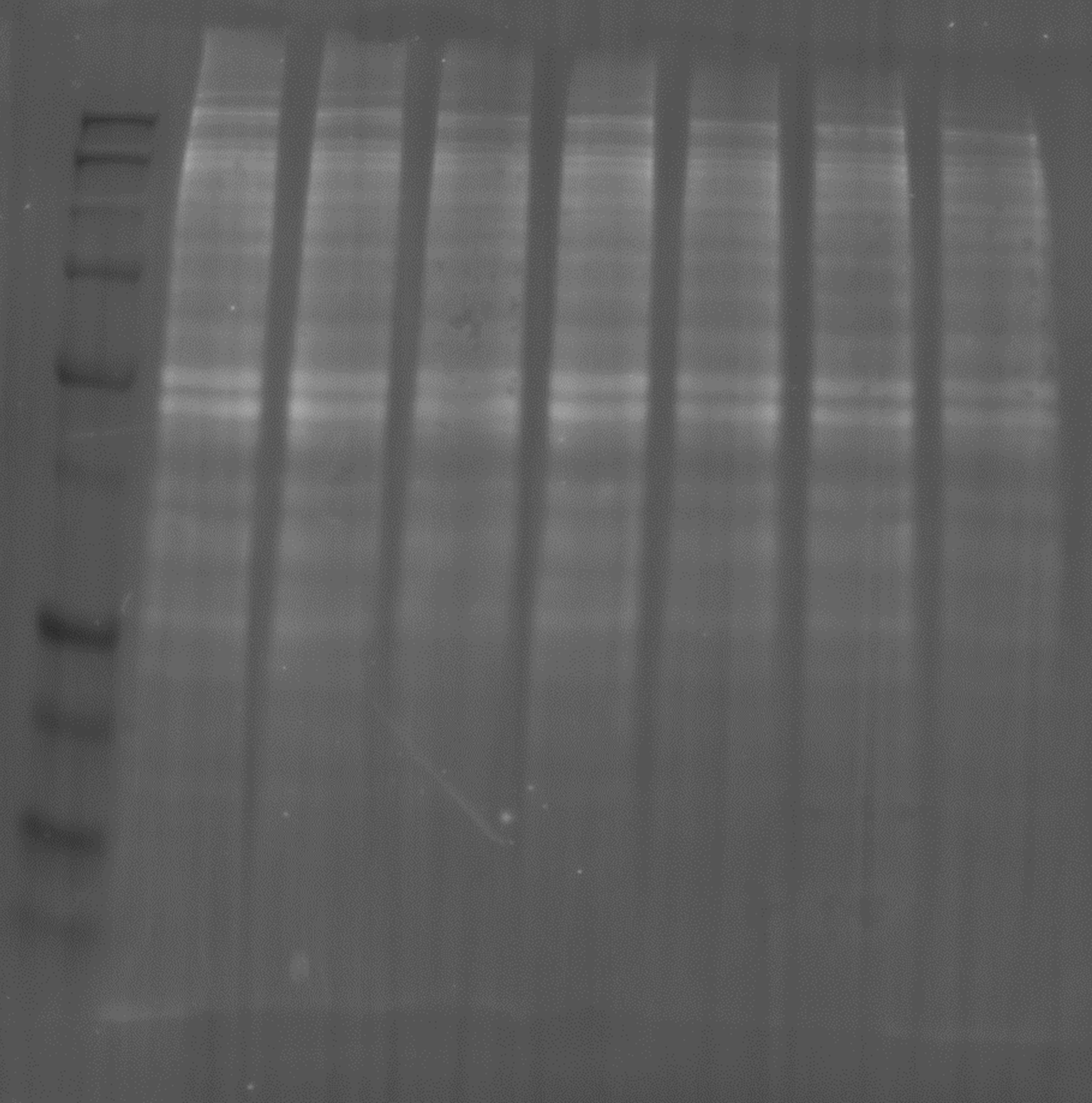

Supplement: S13 Fig — (TIF) [file pcbi.1008202.s014.tif]

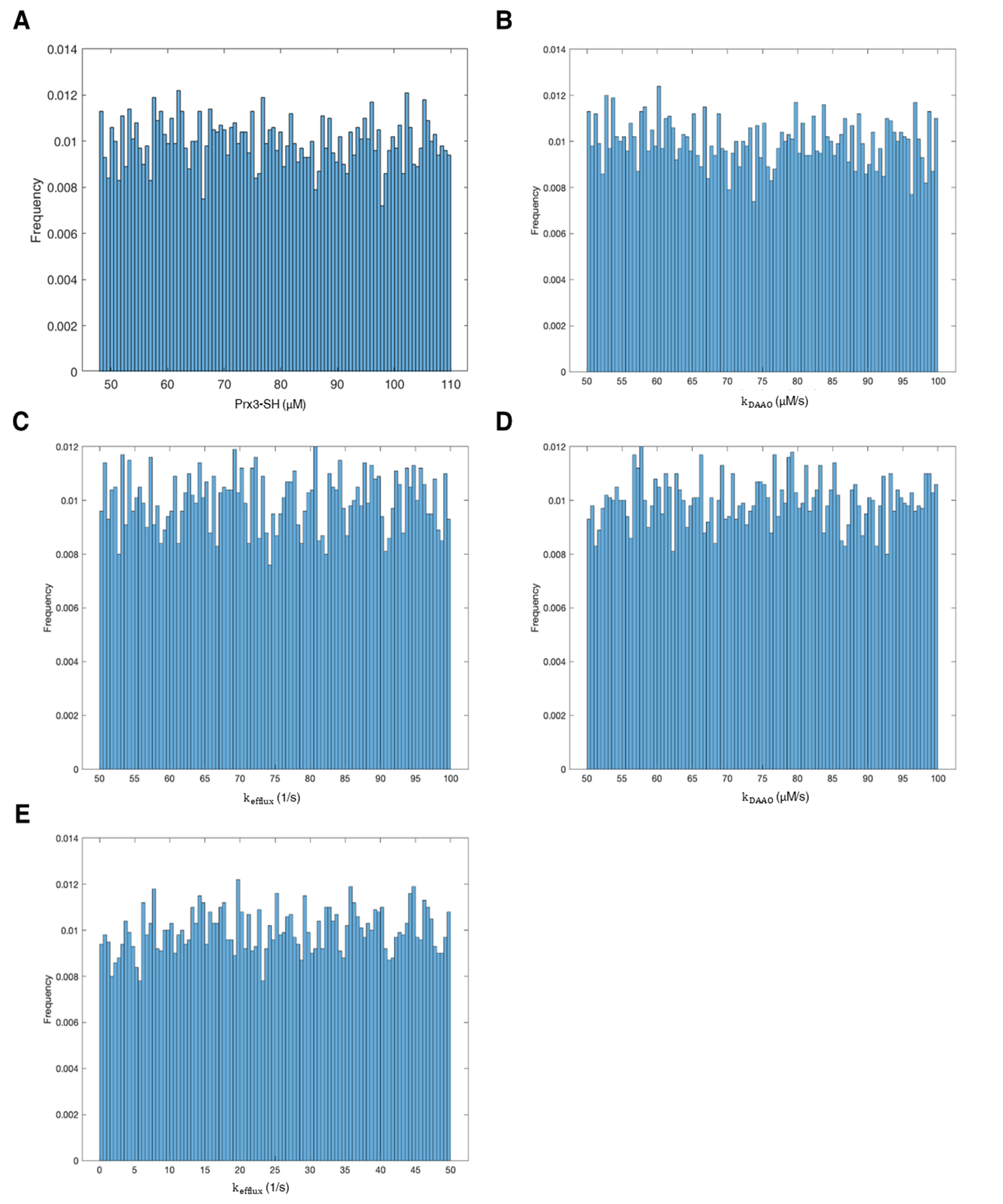

Supplement: S14 Fig — (TIF) [file pcbi.1008202.s015.tif]
